# An Overview of the Sensors for Heart Rate Monitoring Used in Extramural Applications

**DOI:** 10.3390/s22114035

**Published:** 2022-05-26

**Authors:** Alessandra Galli, Roel J. H. Montree, Shuhao Que, Elisabetta Peri, Rik Vullings

**Affiliations:** 1 Department of Information Engineering, University of Padova, I-35131 Padova, Italy; galliale@dei.unipd.it; 2 Department of Electrical Engineering, Eindhoven University of Technology, 5600 MB Eindhoven, The Netherlands; r.j.h.montree@tue.nl (R.J.H.M.); s.que@tue.nl (S.Q.); e.peri@tue.nl (E.P.)

**Keywords:** electrocardiogram, extramural monitoring, heart rate, mechanocardiogram, non-invasive monitoring, photoplethysmogram, sensors

## Abstract

This work presents an overview of the main strategies that have been proposed for non-invasive monitoring of heart rate (HR) in extramural and home settings. We discuss three categories of sensing according to what physiological effect is used to measure the pulsatile activity of the heart, and we focus on an illustrative sensing modality for each of them. Therefore, electrocardiography, photoplethysmography, and mechanocardiography are presented as illustrative modalities to sense electrical activity, mechanical activity, and the peripheral effect of heart activity. In this paper, we describe the physical principles underlying the three categories and the characteristics of the different types of sensors that belong to each class, and we touch upon the most used software strategies that are currently adopted to effectively and reliably extract HR. In addition, we investigate the strengths and weaknesses of each category linked to the different applications in order to provide the reader with guidelines for selecting the most suitable solution according to the requirements and constraints of the application.

## 1. Introduction

The heart is an organ that pumps blood through the blood vessels of the circulatory system. This blood carries oxygen and nutrients to the body, while it removes the metabolic waste [1]. Heart rate (HR) is the rate at which the heart pumps and is measured by the number of contractions (beats) of the heart per minute (bpm) [2]. The HR can vary according to the body’s physical needs, such as the need to use oxygen and expel carbon dioxide, and it is modulated by several internal and external factors including genetics, fitness activity, stress or psychological status, diet, bad habits (e.g., smoking or drinking alcohol), medications, hormonal status, environment, and disease/illness [3].

HR is one of the most important vital signs as it can be considered an indicator of the general health status of a subject. For this reason, already since decades, HR is extensively monitored in critical care areas such as the intensive care unit, post-anesthesia care unit, telemetry monitoring units, and emergency departments where patient conditions can rapidly deteriorate [4,5]. Alarms can be generated when the patient’s HR is outside the normal physiological limits [5]. The American Heart Association states that the normal resting HR in adult humans is 60–100 bpm. A cardiac rhythm higher than 100 bpm at rest is defined as tachycardia, while an HR lower than 60 bpm at rest is defined as bradycardia [6].

Another relevant parameter associated with HR is heart rate variability (HRV). HRV is the variation over time of the period between consecutive heartbeats and is predominantly dependent on extrinsic regulation of HR [2]. Several metrics have been investigated to assess HRV. For example, a standard deviation of the cardiac cycle duration lower than 100 ms is considered unhealthy [7] and could involve adverse events such as sepsis and systemic inflammatory response syndrome in low-weight newborns [8] or an acute inflammatory response in COVID-19 patients [9].

In the last decade, the rapid development of sensor, information, and communication technology has provided tremendous opportunities and challenges in the field of extramural monitoring of health status and well-being [10]. Among others, the HR and HRV are one of the most used physiological parameters for extramural monitoring, as in many extramural situations these parameters can be measured by noninvasive and low-cost technologies. For example, monitoring HRV to estimate athletes’ training (mal)adaptation is becoming normal practice to improve athletic performances [11]. HRV is also used to assess the occurrence of mild or severe dehydration levels caused by intense physical activity or by a specific health status (e.g., old age, cardiovascular or kidney diseases, medications, etc.) [12].

Extramural monitoring opens the way to a prolonged and extended evaluation of the heart rhythm, which has several advantages for both the diagnosis of pathology and the prevention of adverse events related to heart disease. For example, reduced HRV is a strong predictor of mortality in patients with infarction and heart failure; therefore, its continuous assessment helps to recognize patients at risk in order to timely intervene with preventive therapy [13]. Moreover, HRV analysis in extramural settings can be used to indirectly infer arousals associated with obstructive sleep apnea syndrome. This novel diagnostic approach enables both mass screening of the population to efficiently identify those who suffer from this disease and long-term monitoring of patients, which are precluded by the traditional diagnostic approach based on the polysomnogram [14]. Finally, continuous and daily HR monitoring helps to improve users well-being and their awareness of their lifestyle. In fact, HR and HRV provide useful information on stress level [15] and sleep quality [16].

The requirements of HR monitoring devices for remote and out-of-hospital (i.e., extramural) applications are different from the devices used in traditional monitoring. In particular, the needs for minimal obtrusiveness, lightweight, and comfort are crucial. Furthermore, the HR estimation provided by the devices must be accurate, especially in devices that are used for medical and diagnostic purposes, even when measurements are made under challenging acquisition conditions (e.g., high-level noise or motion artifacts and loss of contact) and without the supervision of an expert.

This work presents an overview of the main strategies that have been proposed for noninvasive monitoring of HR in extramural and home settings. In particular, we focus on the sensing principle, which can be classified according to which physiological effect is used to measure the pulsatile activity of the heart. We consider three sensing categories: (i) sensing the electrical activity of the heart, (ii) sensing the peripheral effect of the heart pulse, and (iii) sensing the mechanical activity of the heart. For each of these categories, we focus on an illustrative sensing modality that is presented in detail in terms of sensing principle, strength, and challenges. Electrocardiography is presented as an illustrative modality to sense the electrical activity of the heart. It exploits the dynamic electromagnetic fields generated by the heart. Photoplethysmography is described as an illustrative modality to investigate the peripheral effects of cardiac pulsations. The ejection of blood from the heart into the vascular tree causes changes in blood volume in the microvascular tissue of the peripheral areas. Because blood absorbs light more than surrounding tissues, these microscopic changes in the optical properties of the body surface can be measured by photoplethysmography. Finally, mechanocardiography is presented as a recently developed possibility to describe organ motion and deformation caused by the pulsatile activity of the heart. In fact, at every heartbeat, the pulse wave traveling through the body produces subtle changes in displacements and vibrations of the body surface that can be measured.

In this paper, we describe the physical principles underlying the three categories, the characteristics of the different types of sensors that belong to each class, and we touch upon the most used hardware and software strategies that are currently adopted to effectively and reliably extract the information of interest, i.e., HR and HRV. In addition, we investigate the strengths and weaknesses of each category linked to the different applications. In this way, we provide the reader with a general overview of the strategies commonly used to monitor HR and HRV and we give indications for selecting the most suitable sensing modality in different applications.

## 2. Sensors Based on Electrical Activity

The pumping function of the heart is the result of a rhythmic contraction and relaxation of approximately $10^{9}$ muscle cells [17]. This process is controlled by the propagation of biopotentials through the whole cardiac tissue, culminating in a complex electrical pattern. Heart electrical activity begins through spontaneous depolarization of the sinoatrial (SA) node, which is located above the right atrium. This depolarization propagates through the atrial tissue and is transmitted to the ventricles through the atrioventricular node. From the atrioventricular node, the signal enters the bundle of His. This bundle branches into the tree structure of Purkinje fibers, which provides rapid conduction of electrical signals through the ventricles. The initial stimulus of the SA node thus causes depolarization wavefronts, which ultimately activate the entire mass of the ventricles.

### 2.1. The Electrocardiogram

The heart, from an electrical point of view, can be seen as a dipole. In fact, depolarization and repolarization of cardiomyocytes induce low-intensity electric fields on the surface of the human body. The electrical potential produced by the myocardium is the sum of the potential differences generated by individual cardiomyocytes. These small tensions are recorded through a device called electrocardiograph, which was introduced for the first time in 1903 by Willem Einthoven and Étienne-Jules Marey [18].

The electrical potential generated by the heart’s activity is measured as a potential difference between any pair of electrodes. In non-invasive applications, electrodes are typically made of conductive pads that are attached to the body surface, where the combination of two electrodes forms a lead.

**Figure 1:**
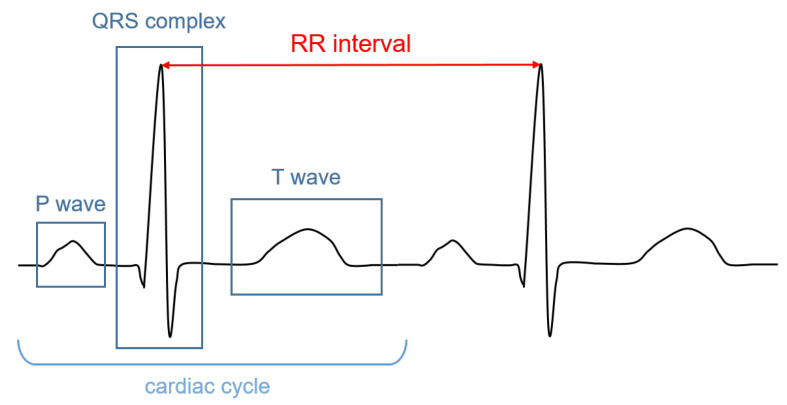
Illustrative ECG signal recorded during one cardiac cycle. The main components are highlighted.

The signal recorded through an electrocardiograph is called electrocardiogram (ECG). It has three main components: the P wave, which corresponds to the depolarization of the atria; the QRS complex; and the T wave, which represent, respectively, the depolarization and the repolarization of the ventricles. The HR (in bpm) can be determined by dividing 60 (seconds per minute) by the RR interval measured in seconds. The RR interval is the distance between two successive R peaks, as shown in Figure 1.

### 2.2. Instrumentation

The acquisition of the potential differences generated by the electrical activity of the heart in extramural and home monitoring settings is based on portable ECG-based HR monitors. These devices are formed by a handheld sensing unit (e.g., a pad, a watch, a band, a necklace, a t-shirt, wireless sensors, etc.) [19,20,21] sensitive to the electrical field and a unit that captures, processes, and displays or transmits the sensed signals.

The interface between the body and the ECG device is formed by the electrodes. To obtain a stable and high-quality ECG signal, these electrodes have the following requirements: (i) the impedance between the electrode and the skin should be minimized to guarantee a signal with high amplitude; (ii) the electrode should provide stable contact with the skin during the acquisition in order to keep contact during motion; (iii) the electrodes must be biocompatible, avoiding adverse reactions of the skin, and comfortable, even when used for a prolonged time.

**Figure 2:**
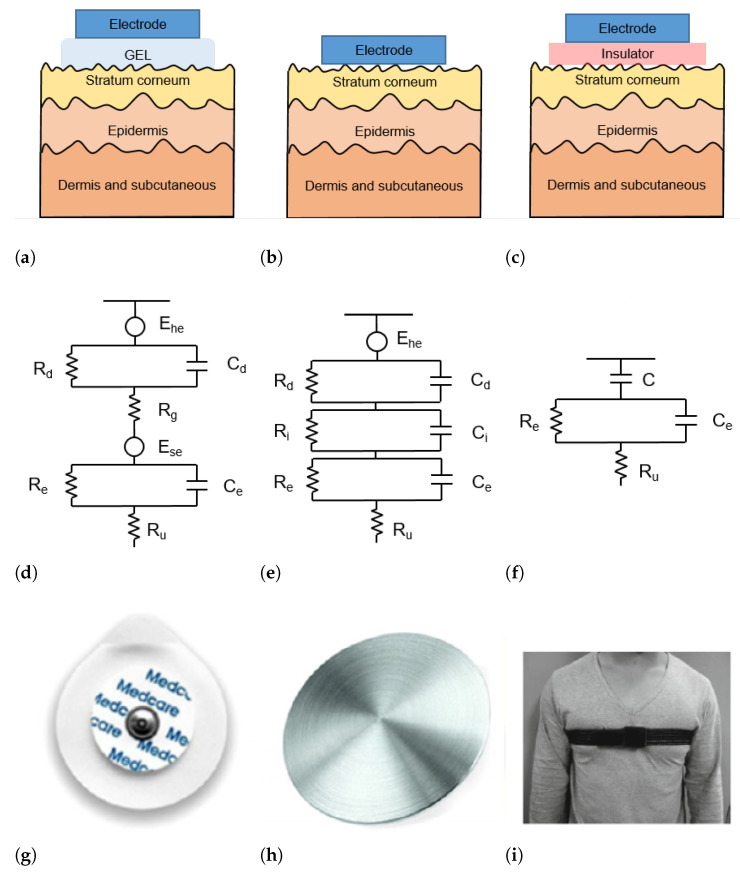
(**a**–**c**) Schematic representation of skin-electrode interface for wet (**a**), dry (**b**), and capacitive (**c**) electrodes; (**d**–**f**) equivalent circuit model of skin–electrode interface for wet (**d**), dry (**e**), and capacitive (**f**) electrodes; (**g**–**i**) example of wet (**g**), dry (**h**), and capacitive (**i**) electrodes.

The skin–electrode interface mainly comprises multiple layers of conductive and capacitive coupling, which are complemented by parallel resistor-capacitor (RC) networks connected in series [22]. Depending on which RC networks are predominant, the type and the operating principle of each sensor are defined. In general, the electrodes used for ECG acquisition can be divided into wet, dry, and capacitive electrodes, as depicted in Figure 2.

#### 2.2.1. Wet Electrodes

Wet electrodes are the standard biosensors for most electrocardiography applications, thanks to the high-quality signals that can be acquired with them. These electrodes are based on an electrode–electrolyte interface, which is characterized by a half-cell potential ($E_{hc}$). The $E_{hc}$ is linked with the impedance of the electrode–electrolyte interface, modeled as the parallel circuit between a resistor $R_{d}$ and a capacitor $C_{d}$. The skin is a dry dielectric surface that creates a barrier for any transfer of charges between the body and the electrode [23]. The electrolyte gel acts as a contact medium and helps transfer charges between the skin and electrode [24]. The resistance of the electrolyte is modeled as the serial resistor $R_{g}$. At the skin level, a difference in ionic concentration across the stratum corneum results in a potential $E_{se}$. The entire epidermis can be considered equivalent to a parallel circuit between a resistor $R_{e}$ and a capacitor $C_{e}$. The impedance of the dermis and subcutaneous tissues can be treated as a resistor $R_{u}$ [25]. The equivalent circuit of wet electrodes is depicted in Figure 2d.

Conventional wet electrodes are silver-silver chloride (Ag/AgCl) electrodes, as illustrated in Figure 2g. A new generation of adhesives allows for the realization of novel electrodes or bio-patches that are less uncomfortable than traditional ones [26]. Although contact electrodes provide good signal quality, they require skin abrasion to minimize the skin impedance and can cause adverse reactions (e.g., allergic reactions and skin irritation) especially if the electrodes are worn for a long time. In addition, the quality of the acquired signal decreases with dehydration of the gel as a result of prolonged use [27].

#### 2.2.2. Dry Electrodes

Dry electrodes are constituted by metal discs (such as that shown in Figure 2h) or conductive plastics and are in direct contact with the skin, without the need for any gel. Therefore, the resistance $R_{g}$ in Figure 2d is replaced by a parallel circuit of a capacitor $C_{i}$ and a resistor $R_{i}$. The schematic circuit and an example picture of a dry electrode are shown in Figure 2b,h, respectively. During the employment of dry electrodes, sweat that is accumulated at the skin surface can chemically resemble the electrolyte solution in wet electrodes and thus act as a substitute for the gel [28].

Dry electrodes are proposed to overcome the issues of wet electrodes related to long-term applications, which are common in extramural HR monitoring. Dry electrodes are expected to satisfy long-term HR monitoring requirements because they do not require skin pretreatment and conductive gel, and can be manufactured with advantages such as good stretchability, portability, small size, and low cost. In fact, recent advances in research have opened the way to printable and flexible dry electrodes [29], making them more comfortable than traditional wet electrodes, without incurring adverse reactions. In addition, dry electrodes can maintain good contact with the skin, even during intense motion, such as fitness activity, and are suitable for prolonged use. These qualities make them especially suitable for ambulant applications [25]. Conversely, they have a higher equivalent impedance and, compared to signals acquired with wet electrodes, signals received by dry electrodes are characterized by lower amplitude and hence lower signal-to-noise ratios (SNR). Therefore, many studies conducted in recent years aim to overcome the shortcomings of low SNR to enable reliable HR and HRV monitoring with dry electrodes.

In the dry electrode manufacturing process, researchers mostly choose to combine conductive materials with flexible substrates (e.g., polydimethylsiloxane (PDMS), polyimide (PI), polyethylene terephthalate (PET), etc.) by lithography, sputtering, deposition, blending, electrostatic spinning, electrostatic spraying, chemical coating, and screen printing [30].

Conductive materials used for dry electrodes include metals and derivates (e.g., silver (Ag), silver nanowires (AgNWs), gold (Au), and titanium nitride (TiN)), which are selected because of their excellent electrical conductivity. For example, Zheng et al. [31] proposed an ECG monitor for wearable applications based on two sensor patches. The sensing unit is realized by means of ultrathin (19 μm) Ag/PDMS thin-film electrodes using a coating process, which enables a skin contact impedance comparable to that of commercial wet electrodes. Due to its ultrathin properties, the PDMS film conforms very closely to the curved surface of the skin, maintaining stable contact for 24 h. However, metallic materials are not widely used for dry electrodes due to their high cost, combination issues with flexible substrates, and the long time required for manufacturing [25].

Because of their excellent electrical conductivity and stability, carbon materials (e.g., carbon, carbon black (CB), graphene, carbon nanotubes (CNTs), carbon nanofibers (CNFs), etc.) have a wide range of applications in the field of bioelectric dry electrodes. Kim et al. [32] proposed a novel flexible electrode based on the gecko-inspired hierarchical microstructure using a mixture of CNT and graphene. Such an electrode has self-absorption ability, which is crucial for wearable applications because fixing the electrode with a tape or elastic strap is not desirable. The proposed electrode showed good cyclic adhesion and its conductivity, during movement, did not show a significant decrease, satisfying the requirements for long-term ECG measurement. Furthermore, since the materials used are super-hydrophobic, this electrode is well-suited for a reusable, sustainable, and low-cost ECG monitoring system. Although carbon materials are currently the most widely used materials for dry electrodes, there are still some improvements that can be made in terms of reducing the harm of CNTs to the human body and making the manufacturing process easier through technological improvements [25].

Although metallic and carbon materials have good conductive properties, conductive polymers outperform them in terms of biocompatibility and chemical stability. One of the most widely used polymers is poly 3,4-ethylenedioxythiophene:polystyrene sulfonate (PEDOT:PSS) because it has excellent mechanical properties, high-temperature resistance, and good biocompatibility. It can be used on the skin surface to enhance electrical conductivity and reduce contact impedance. PEDOT:PSS can be easily processed into films using simple techniques such as inkjet printing and has recently been successfully integrated into textiles (e.g., cotton, polyester, nylon, etc.) [33,34] or paper-based materials [35]. For example, Sinha et al. [36] fabricated a textile electrode using screen printing technology, which was directly integrated into sports tights. However, the high skin-electrode contact impedance due to low contact pressure and unstable contact reduces the quality of the acquired ECG signals. Yet, the electrode is still acceptable for HR-monitoring purposes. Integrating electrodes, wires, and other modules within smart clothes could improve the sustainability and convenience of HR monitoring. However, the decrease in signal quality after repetitive washing is a flaw that needs further investigated [37].

#### 2.2.3. Capacitive Electrodes

The capacitive sensors do not require direct contact with the skin. In fact, a thin insulator material is placed between the metal electrode and the skin [24] (Figure 2c). Charge transfer from the skin to the electrode takes place by means of capacitive coupling at the skin-dielectric and dielectric-electrode interface [38], represented as *C* in the equivalent circuit in Figure 2f.

The most common materials used as dielectrics are conductive rubber [39] and biomedical e-textile. In Figure 2i a t-shirt is placed between the electrode and the skin. Capacitive electrodes can be incorporated into everyday usable garments and objects. For example, Lim et al. [40] and Baek et al. [41] proposed capacitive electrodes embedded in an office chair so that the ECG can be acquired simply by sitting on it. Uguz et al. [42] installed capacitive electrodes in a car seat to measure the HR of the user during driving. Nemati et al. [38] embedded their electrodes in a t-shirt and Varadan et al. [43] in a bra.

Capacitive electrodes are comfortable and suitable for long-term monitoring because they do not cause skin irritation. On the other hand, the lack of gel between the skin and the electrode increases the contact impedance, resulting in a reduced signal amplitude [27]. Furthermore, the presence of the insulator material involves a large discrepancy in terms of shape between the actual physiological signal and the signal obtained through capacitive electrodes [30]. Furthermore, due to the lack of direct contact, the ECG signals acquired with capacitive electrodes are highly sensitive to movements, producing ECG signals that are strongly corrupted by artifacts [22].

### 2.3. Signal Processing Approaches to Extract the HR

Once the signals have been acquired by the sensing unit, the ECG samples are transmitted to a processing unit (e.g., a smartphone, a smartwatch, a cloud environment, etc.) [44,45], where they can be processed in real time or stored for later processing. Late processing occurs in Holter devices, which are employed especially to diagnose specific cardiac pathology and follow-up cardiopathic patients. In contrast to the delayed processing in Holter devices, fitness trackers, and devices that can generate alarms for emergency assistance typically require real-time analysis of the acquired data.

Most of the methods proposed in the literature to estimate HR from ECG signals are based on the detection of R peaks. Over the years, several methods based on different strategies, such as wavelet transformations, filtering, machine learning, empirical mode decomposition, Markov models, etc., were proposed.

**Figure 3:**
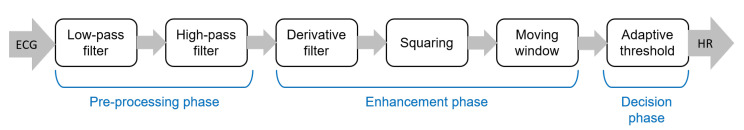
Main steps of the well-known Pan–Tompkins [46] algorithm to detect R-peaks.

The pipeline of algorithms for R-peak detection can be summarized in three main steps: (i) preprocessing aimed to suppress noise and artifacts; (ii) enhancement of the QRS complexes, reducing at the same time the amplitude of the other waves in the ECG signal in order to make the detection of the R-peak more reliable; and (iii) R-peak detection based on a decision rule approach. The most popular approach for the detection of R peaks is the well-known Pan–Tompkins algorithm [46], which was proposed in 1985 and is still widely employed. The block diagram of this algorithm is reported in Figure 3.

Although the Pan–Tompkins algorithm was proposed several decades ago, it is still widely used because it enables efficient detection of QRS complexes, satisfying requirements related to noise rejection, computational load, and accuracy. In fact, the combination of low computational load and high accuracy makes this approach suitable for real-time applications.

ECG signals acquired in extramural and home settings often have a lower quality than signals acquired in a hospital setting. In part, this can be explained by the fact that for long-term extramural monitoring, often dry or capacitive electrodes are used. As mentioned above, these electrodes result in low-amplitude and distorted signals. Furthermore, the acquisition of the ECG during daily life results in signals affected by a large number of motion artifacts [47]. Therefore, there is the need to design ad hoc denoising algorithms aimed at sufficiently increasing the SNR to allow the detection of R-peaks with high accuracy. For example, Peng et al. [48] employed a discrete wavelet transform (DWT) with a moving average to enhance the SNR of ECG signals that were acquired using capacitive electrodes placed over a t-shirt. DWT was also used by Kota et al. [49] to remove noise from signals acquired during physical activity with dry electrodes. Galli et al. [50] presented a denoising algorithm based on a compressive sampling Taylor–Fourier multifrequency (CSTFM) approach, which enables the effective removal of the superimposed noise from ECG signals acquired by low-cost and wearable smartphone-based devices.

Additional sensors (e.g., triaxial accelerometers, multiple biopotential sensors for multiple ECG leads, and electromyography) can also be embedded in portable ECG devices to effectively remove noise. As an example, multiple leads enable the design of a denoising approach based on array processing methods, such as by Lazaro et al., who used Principal Component Analysis (PCA) combined with normalized least mean squares (NLMS) adaptive filtering to effectively reduce the EMG noise from the ECG channels [51].

### 2.4. Extramural Applications

Handheld ECGs involve the use of dry electrodes (e.g., pads, bands, conductive fabrics integrated into garments) [20,52], or capacitive electrodes embedded in furnishing accessories (e.g., pillows, beds, blankets) [53,54]. To minimize the obtrusiveness of the device, the number of leads used is commonly limited to one. Furthermore, the position of the electrodes is not always standardized; when the electrodes are for instance embedded in objects, the position of the electrodes with respect to the body is quite common [53].

The applications of portable HR monitors based on ECG are many. For instance, wearable ECG bands (e.g., QardioCore (Qardio®, San Francisco, CA, USA)) or adhesive patches (e.g., Zio (iRhythm San Francisco, CA, USA)), are widely employed to track fitness activity [55]. To obtain the most effective fitness workout, the HR should be kept within the optimal boundaries [44]. Monitoring HR and HRV during physical exercise is an important aspect of both sports and rehabilitation medicine because high levels of training or high-performance sports entail a high degree of stress for the human heart that could lead to abnormal or undesired behavior in some athletes. On the other hand, HR monitoring can also be used to quantify the calories burned during exercise [21].

Wearable ECG monitors are also helpful in telemedicine scenarios. For addressing a cardiac rehabilitation condition, Worringham et al. [56] presented a *e*-Health network consisting of a smartphone, an ECG, and a GPS-based system to remotely monitor the exercise of patients. The system provided a more flexible way to remotely perform unsupervised cardiac rehabilitation, where HR information was transmitted by a programmed smartphone to a server where data could be monitored in real time by qualified medical personnel. In a similar scenario, Baig et al. [57] discuss a system for monitoring the HR of elderly living in remote locations based on wireless textile electrodes. El Attaoui et al. [58] presented a remote ECG monitoring system that can evaluate cardiac electrical activities and detect HR and HRV disorders in patients who suffer from chronic heart disease (e.g., strokes and heart attacks) in order to reduce hospitalizations for examination and follow up. Wearable, wireless, and mobile monitoring systems can provide the best possible solution for telemedicine because they address convenience and comfort; they reduce costs, time, and travel, and enable immediate medical assistance in case of an emergency.

Moreover, by exploiting continuous monitoring, which is made possible by the advent of portable technologies, a higher number of pathologies related to heart rhythm can be diagnosed, especially intermittent diseases such as atrial arrhythmias [59].

### 2.5. Future Developments

Although ECG-based HR monitoring is widely used in both clinical and extramural settings and several commercial devices are already available on the market, some improvements can still be made. In particular, such improvements include more economic and sustainable manufacturing processes. In fact, the cost associated with the manufacturing of comfortable sensors and ECG devices, as well as the number of waste products (e.g., disposable electrodes), are very high. Therefore, future developments in this field will be oriented to the development of prolonged use and reusability of electrodes in order to reduce both costs and environmental impact.

## 3. Sensors Based on Peripheral Effects of Heart Activity

Due to the pumping function of the heart, the blood volume and the blood pressure at a given location in the body will vary over time. Given the elastic properties of the vessels, this change in blood pressure causes the arteries of the peripheral system to vary in size. The change in size allows the local blood volume to temporarily vary. As the blood is pumped by the heart, the volume of blood at a certain location will increase and decrease, showing a pulsatile behavior. This pulsatile behavior is delayed compared to, e.g., the ECG as the pulse needs to propagate through the arteries. In fact, this delay, typically referred to as the pulse transit time, is hypothesized to be related to blood pressure variations [60].

### 3.1. Photoplethysmography

**Figure 4:**
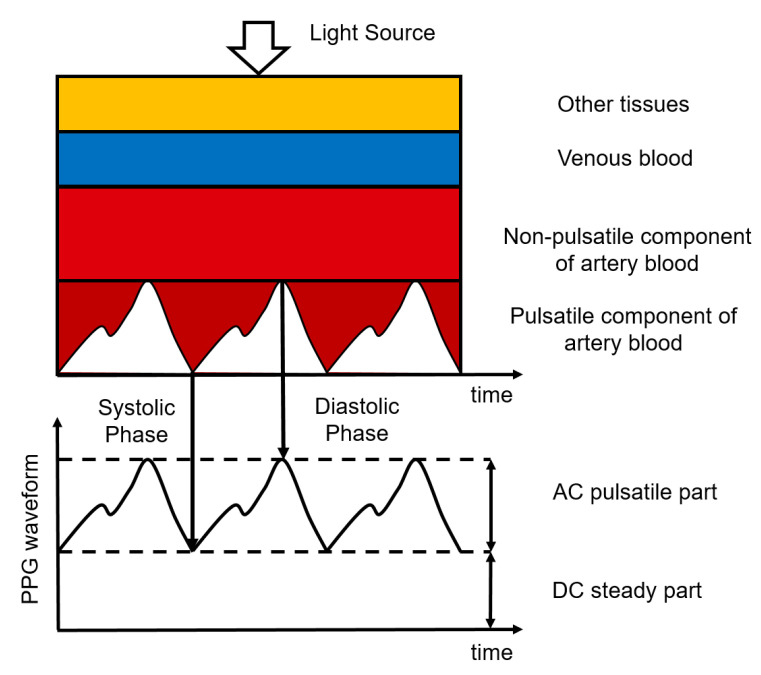
Composition of the PPG signal where different tissue layers cause the absorption of light. Figure reproduced from [64].

Photoplethysmography (PPG) is an optical measurement technique, used to detect blood volume changes in the skin [61]. It uses a sensor composed of a light-emitting diode (LED) to emit light of a certain wavelength and a photodetector to detect the light transmitted through or reflected by the skin, typically making the hardware for PPG simple and cheap. The sensor can be placed unobtrusively on the skin surface, where it can remain for a long time. The intensity of the light detected by the photodetector is affected by the amount of blood in the pathway of the light. This amount varies due to the pulsatile blood volume changes in the artery [62]. When there is more blood in this pathway, the absorption of light is higher and the intensity measured by the photodetector is lower. Most of the light absorption is caused by the non-pulsatile part of the arterial blood, the venous blood, and other tissues. The PPG waveform, therefore, has a large DC component and only a small AC component. The ratio between the AC and the DC components of the PPG waveform is called the perfusion index and is related to the blood perfusion [63]. This principle is illustrated in Figure 4.

The absorption of the emitted light depends on the wavelength used due to the different absorption coefficients of the tissue for various wavelengths [65], in accordance with the Beer–Lambert law [66]. Indeed, absorption of longer wavelengths (e.g., red and near-infrared light) is lower, meaning they will penetrate the tissue deeper, up to the larger arterioles and possibly arteries in the deep dermis [67]. In contrast, shorter wavelengths (e.g., green light) penetrate less deeply into the tissue and cannot reach the arteries, where the major pulsatile component is present. Variations in the intensity of detected light recorded with shorter wavelengths originate from changes in the density of the capillaries, as suggested by Kamshilin et al. [68]. In fact, when blood volume increases, the size of the arteries will also increase due to their elastic properties, resulting in a compression of the tissue located between the arteries and the skin. Consequently, absorption and scattering coefficients will reflect the underlying cause of the variations and will therefore show the same pulsatile differences in light absorption. This hypothesis is further supported by the finding that HR can be determined with green light as accurately as it can be with a red light [67].

**Figure 5:**
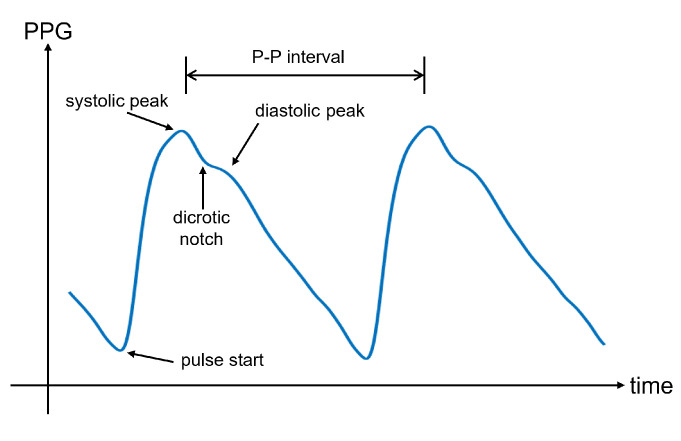
An illustrative PPG wave for one pulse, with the pulse start, systolic, and diastolic peaks, the dicrotic notch, and the pulse to pulse (P-P) interval indicated. The P-P interval is the time between to consecutive systolic peaks. Note that with respect to Figure 4, the signal is flipped along the vertical axis, to represent the volume instead of the detected light intensity.

In Figure 5, an illustrative PPG wave is shown. The representation of the PPG wave is the inverted sensor readout to express blood volume rather than light intensity. Indeed, less light recorded by the photodetector corresponds to a higher amount of blood in the arteries, which is caused by the blood injected into the arteries during systole. This phase is responsible for the systolic peak in the PPG wave. However, the reduction in blood volume during diastole involves a decrease in absorbed light. This downward trend can show a separate peak, called the diastolic peak, which is the result of the reflections of the pressure wave [69]. The local minimum that occurs between the systolic and diastolic peaks is the so-called dicrotic notch, which represents the transition from the end of systole to the start of diastole [70]. Many physiological parameters can be extracted from the PPG wave, including the systolic amplitude, pulse width, pulse area, peak-to-peak interval, and pulse interval. An extensive overview of the PPG parameters can be found in [71].

The amplitude of the PPG signal is influenced by many controllable factors, such as sensor geometry, emitted light intensity, and photodiode sensitivity, but also due to uncontrollable biological factors, such as the thickness of the skin at the measurement location, the skin color, and the tissue composition at the measurement location [72]. This implies that the PPG signal is not straightforwardly comparable between different users and even between different measurements within the same user. Therefore, it is desirable to consider ratios between amplitudes rather than absolute values.

### 3.2. Instrumentation

The sensing unit consists of two parts: the light source and the photodetector. The light source is an LED. To increase the amount of light detected by the photodetector, an array of LEDs can be used. This is because the path of light can fluctuate heavily during recordings, especially due to the movements. An array of LEDs can then be exploited to reduce these motion artifacts.

**Figure 6:**
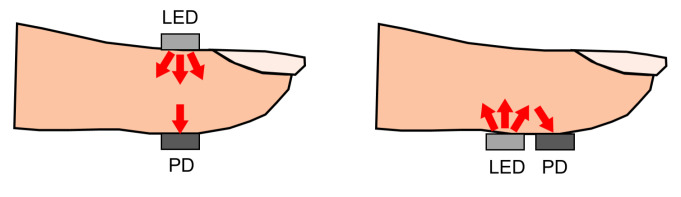
Schematic overview of transmissive (**left**) and reflective (**right**) PPG using an LED and a photodetector (PD). For the transmissive PPG, LED and PD are placed on opposite sides of the finger. For reflective PPG, the LED and PD are placed on the same side. Figure reproduced from [64].

PPG sensors can operate in two modes: transmissive and reflective PPG. Transmissive PPG is the mode in which the photodetector is opposite to the LED. In this case, the light travels from the led directly through the tissue before it reaches the photodetector. If the path length of the light is too large, almost all of the light will be absorbed before reaching the detector. For this reason, measurement locations are limited to the fingertip, nose, earlobe, or toe. Too much pressure on the measurement location due to sensor placement can also suppress variations in peripheral blood volume, which may result in a reduction in signal oscillations [73]. When using reflective PPG, the LED, and the photodetector are placed on the same side of the tissue. This configuration is convenient for sensor design and can also be used when the underlying tissue introduces a significant absorption coefficient in the light path in the case of a transmissive PPG sensor design. This happens when the distance between the LED and the photodetector is too large, or when the measurement location does not have a larger artery present. A visual representation of transmissive and reflective PPG can be seen in Figure 6.

Sensor hardware for PPG measurements is often relatively minimal. Besides the light and the photodetector, the hardware typically consists of a few components: a signal amplifier and some basic signal filters. Because the light intensity of the received is very low, the corresponding voltage difference at the output of the photodetector is very low. For this reason, the voltage is amplified. Additionally, the information in a PPG signal is mostly low frequency (less than 5 Hz); often a sampling frequency between 125 and 500 Hz is chosen. The low frequency content in the PPG is used for the definition of bandpass filters, with the aim of removing DC and high frequency components from recorded data. Depending on the final application, more strict constraints for signal filters or sampling frequency are common to sample the signal. For example, to determine the average HR, Béres et al. [74] showed that the sampling frequency can be lowered to as much as 5 Hz, while for accurate HRV analysis, a sampling frequency of 20 Hz with the addition of interpolation is enough.

One of the sources of noise in PPG signals is other variations in the illumination of the measured tissue or photodetector that are not caused by changes in blood volumes but, for instance, by changes in the ambient light. To reduce the amount of variation in illumination caused by ambient light, the LED and the photodetector are typically shielded from outside light sources. Another important source of noise is caused by motion, i.e., motion artifacts. The choice of the light source, specifically the wavelength/color of the light, can help reduce this type of noise; shorter wavelengths have been shown to be less susceptible to motion artifacts [75].

### 3.3. Signal Processing Approaches to Extract the HR

The peak-to-peak interval, which is the time between two systolic peaks, is commonly used to determine HR, as these peaks are the most easily detected feature of the PPG signal. Using this method, HR can be determined directly in the time domain. A more robust way of detecting HR is based on frequency-domain analysis [76]. Here, the main component of the obtained Fourier series (besides the large DC component, if it has not been filtered out) is the frequency of the heartbeat. However, HR estimation in the frequency domain relies on window-based frequency analysis and hence only provides trends in HR, instead of beat-to-beat variations. Due to the increased difficulty in accurately measuring smaller variations of heart rate in the frequency domain, post-processing strategies can often be included for more accurate results, such as the Kalman filtering proposed by Galli et al. [76].

When HR detection is performed in the time domain, the detection of the peaks is affected by the noise in the data. This noise can be partially suppressed by digital (bandpass) filtering techniques. Unfortunately, finding the optimal trade-off in the design of these filters is a challenging task, as excessive filtering can distort the pulse shape, but too little filtering can result in the quasi-DC component dominating over the AC pulse [77], complicating peak detection. The implementation of bandpass filters can be completed in several ways. For instance, Chatterjee et al. proposed to use a combination of a Butterworth IIR filter and a Savitzky–Golay FIR filter [78]. An extensive overview of different state-of-the-art signal processing techniques to determine HR and HRV in wrist-worn PPG data, and their performance with respect to the gold standard, ECG, can be found in [79].

Even though the choice for short wavelengths reduces the presence of motion artifacts in PPG data, these artifacts are still the dominant interference. The main challenge in HR extraction from PPG signals is therefore related to the motion artifacts [80]. In fact, the relative movement between the tissue and the sensor modifies the path of the light, distorting the intensity of the light that falls onto the photodetector and hence affects the acquired signal. Data containing a large number of motion artifacts is often unusable. To detect segments of the signal affected by motion artifacts, several signal processing strategies have been proposed. A signal quality index has been used to describe the presence of noise and artifacts in short PPG segments [81] and more recent studies have used neural networks to detect and exclude segments that are contaminated by motion artifacts from further processing. Goh et al. [82] used a 13-layer one-dimensional convolutional neural network and reached an accuracy of 94.5% in classifying signal quality.

Despite the good performance achieved in quantifying signal quality, low-quality recordings still entail a significant impediment for various applications. When low-quality segments are discarded from processing to extract the HR, the monitoring is non-continuous. Whereas for some applications this can be acceptable, for others it is not. For these situations, some researchers focus on the removal of the motion artifacts. As described by Pollreisz et al. [83] other sensors might be included to provide more information about the motion, such as an accelerometer. Lai et al. [84] reported a mean average percentage error with respect to the HR as recorded by the ECG of 2.57% during heavy exercise, using the accelerometer to provide additional information, whereas Hara et al. [85] used a second PPG sensor located a short distance from the skin for extra information on motion artifacts, and achieved a root mean squared error (RMSE) of 7.1 bpm during rest, walking and jumping.

### 3.4. Extramural Applications

Due to the ease of use and comfort provided by PPG sensors, PPG is often the technology chosen for applications that require prolonged HR and HRV measurement. Examples of such applications are sleep monitoring [86] and detection of paroxysmal irregular heart arrhythmia [87]. PPG is also used in commercial applications to track HR and HRV during exercise. Smartwatches aimed at determining the heart rate often have reflective PPG integrated with green light [88]. The reason that most smartwatches are based on PPG with green light stems from the fact that, on the one hand, the use of smartwatches, especially during exercise is associated with increased levels of motion, while, on the other hand, as mentioned before, smaller wavelengths such as green are less susceptible to motion artifacts.

Besides measuring the heart rate, PPG is also used in a variety of other applications. One of the most common ones is oxygen saturation [89], but PPG can also be used to determine a variety of biological markers that are linked with the timing differences between distinctive features of the PPG signal or the pulse morphology (e.g., the time between the systolic and diastolic peak or the presence of the dicrotic notch). The relationship between these features and the biological factors affecting them is an active field of research. For example, the dicrotic notch shape is known to deteriorate with age. Furthermore, Evans [90] used PPG morphology to determine blood vessel vasoconstrictor activity, Jönsson et al. [91] determined ankle pressure, Mansi et al. [92] determined cold sensitivity, and McCombie et al. [93] studied cardiac output. In combination with ECG, PPG can be used to determine the Pulse Transit Time (PTT), which is the time between the start of the heartbeat and the start of the pulse. This PTT has been demonstrated to be a proxy for blood pressure [94].

### 3.5. Future Developments

PPG has been shown to enable determining HR and HRV, reaching performances similar to ECG-based HR under specific conditions [79]. Performance rapidly degrades when motion artifacts are introduced [95]. In an extramural setting, the removal of motion artifacts remains of critical importance for the development of PPG-based solutions. Because PPG allows for long-term unobtrusive measuring, applications for monitoring changes in user condition, detecting diseases or monitoring sleep cycles show many potentials. An advantage of sleep monitoring is the relatively low number of motion artifacts.

PPG can also be obtained without contact of sensors with the skin (also called remote PPG) [96,97]. Such a technique is based on a video that records the reflection of ambient light by the skin. The advantage of this type of PPG is that the recorder can be up to several meters away from the subject, minimizing the obtrusiveness of the solution. On the other hand, the distance between the subject and the recorder is responsible for a significant reduction in the SNR. Indeed, the intensity of the light that is detected will be reduced, and noise sources, such as varying background illumination, will increase. This produces variations in the detected signals that do not depend on the pulsating blood. The reduction in SNR makes the ability to obtain the heart rate reliably harder as compared to contact PPG. When a user is moving around in an extramural setting, additional tracking is required and even more noise is introduced. However, the method is extremely non-invasive, as it involves no wearable sensors. The prevalence of phones and other devices capable of recording video also makes it a widely available solution. The technique has been shown to have a lot of potential in sleep monitoring [86,98]. Sun et al. [99] used remote PPG to monitor the heart rate during exercise, where they have a stable video camera, reducing the motion artifacts from a moving sensor. McDuff et al. [100] used remote PPG to determine the systolic and diastolic peaks. Despite the large potential for video-based PPG, in the case of extramural measurements, the recording is much less controlled, typically leading to a further reduction in the SNR compared to video-based PPG in controlled settings such as the hospital. Therefore, before video-based PPG can be used in real applications, further steps need to be taken to make this type of measurement more reliable.

## 4. Sensors Based on Mechanical Activity

It is a long-known fact that each heartbeat imparts movements to the entire body when blood is ejected into the arteries [101]. Ejection of blood through the descending aorta produces a backward recoil movement that is correlated with heartbeat. Local movements induced by mechanical beating of the heart also propagate to the skin surface of the chest wall. The mechanocardiogram (MCG) is a graphical representation of these movements.

### 4.1. The Mechanocardiogram

The MCG is a container for all measurements of mechanical vibrations that are caused by cardiac motion. It can be subclassified by taking into account the type of sensors that are used and whether the detected motion is localized on the chest wall or distributed throughout the body. The ballistocardiogram (BCG) describes motions distributed over the whole body. Accelerations and rotations of the chest wall are described by seismocardiogram (SCG) and gyrocardiogram (GCG), respectively.

The MCG was investigated through the 1900s [102,103] but was initially largely neglected by the medical community [104]. With technological advancements that simplified the acquisition of these signals, MCG is gaining new attention in research in recent decades. Such research has not yet been translated into commercial devices.

**Figure 7:**
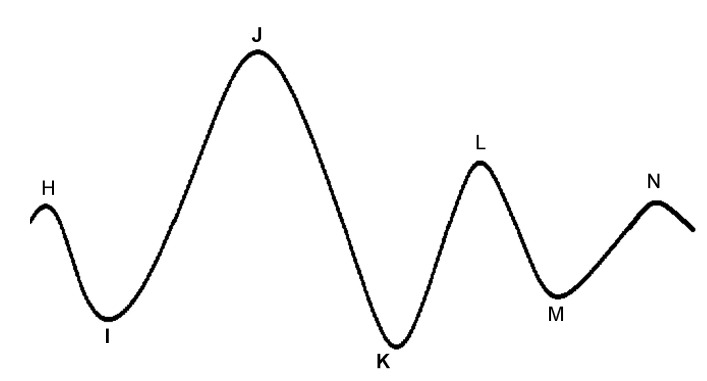
A characteristic ballistocardiogram waveform for one heartbeat. Each peak is denoted with letters H-N. The most relevant peaks related to physiological functions are denoted as I, J, and K.

The BCG is characterized by several waves with I, J, and K being the most visible, as shown in Figure 7. Early studies have shown correlations between BCG measurement alterations and cardiovascular functions [105,106,107]. Although the exact mechanism of the genesis of the BCG waveform and its physiological origin are not as well understood as ECG, several pertinent studies have been published that advanced in this direction. Kim et al. [108] proposed a mathematical model that gave meaning to the timing and amplitude of I, J, and K waves, where the time interval between I and J was hypothesized to represent the aortic pulse transit time; the amplitude of J wave was hypothesized to indicate relative changes in the aortic pulse pressure; the ratio of the amplitude of the J-K down-stroke to the amplitude of the J wave was hypothesized to indicate pulse pressure amplification. Guidoboni et al. [109] proposed another mathematical model that was shown to have captured the fundamental cardiovascular mechanisms which generate the BCG waveform. The predominant features of the BCG waveform captured by this model were reported to be capable of predicting systolic failure.

**Figure 8:**
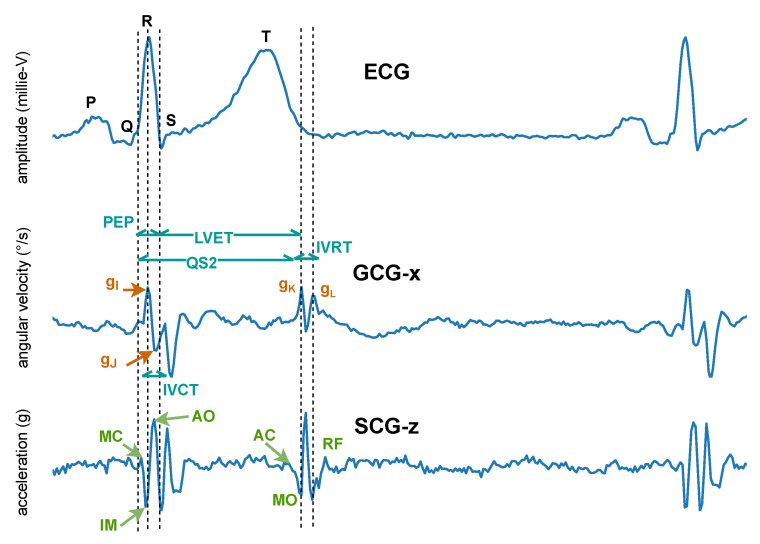
Characteristic seismocardiogram (SCG) and gyrocardiogram (GCG) measurements from the sternum, co-currently collected and annotated according to the ECG. Inside the SCG waveform, the identified systolic events include mitral valve closure (MC), isovolumic movement (IM), aortic valve opening (AO), rapid systolic ejection (RE), and aortic valve closure (AC). Identified diastolic events include mitral valve opening (MO) and early rapid filling (RF). Inside the GCG waveform, the $g_{I},g_{J},g_{K}$, and $g_{L}$ points of the waveform along the x-axis of the GCG are annotated and used to estimate cardiac time intervals, including isovolumetric contraction time (IVCT), isovolumetric relaxation time (IVRT), the total electromechanical systole (QS2), the left ventricular ejection time (LVET), and the pre-ejection period (PEP) [115].

In 1957, instead of measuring the impact of the heart’s mechanical activity on the whole body, Mounsey et al. [110] acquired accelerometer-based BCG from the human chest, which was later renamed seismocardiogram by Baevskii et al. [111] in 1964. Unlike BCG, which measures the whole body’s recoil movement as a reaction to general cardiovascular forces (i.e., heart beating and blood ejection) and thus can be detected all over the body, SCG measures to a large extent local linear acceleration perpendicular to the chest [110] or local linear accelerations along the abscissa, ordinate, and applicate (i.e., x-, y-, and z-axes) [112,113]. A characteristic SCG measurement collected in dorso-ventral direction (i.e., z-axis) is shown in Figure 8. Its waveform appears very similar to BCG. However, as reported by Migeotte et al. [114], there are some differences in timings and amplitudes of the waves and BCG shows smoother curves and lower frequency content than SCG.

In 2015, Meriheinä et al. [116] proposed to measure the cardiac activity manifesting as rotational movements on the chest. In 2017, it was named gyrocardiogram by Tadi et al. [115] who obtained a three-dimensional measurement on the sternum, where the x-axis is horizontal across, and the y-axis is vertical across and the z-axis is perpendicular to the front torso, respectively. Their experimental results demonstrated that GCG is less sensitive than SCG to intra-subject and inter-subject variability in signal morphology and has higher SNR, which coincides with the study of Migeotte et al. who showed that 60% of cardiac vibrational energy is contained in the gyration signal [117]. A characteristic GCG measurement along the *x*-axis is shown in Figure 8.

### 4.2. Instrumentation

The similarities and differences in the sensing modalities for the acquisition of BCG, SCG, and GCG are summarized in Table 1. In this section, we focus on contact-based sensing modalities, as they are widely investigated in the literature. Some initial studies on contact-free solutions are also present and are discussed as future developments in Section 4.5

Sensors for MCG measurements do not require direct skin contact, eliminating potential skin hazards that can be present when applying ECG electrodes or contact-based PPG. However, these sensors are more susceptible to motion distortions than ECG and PPG.

When MCG is recorded using inertial sensors (i.e., accelerometer and gyroscope), the cardiac signal is typically represented by using the z-axis of the three-dimensional SCG/BCG measurement. For three-dimensional GCG measurements, the x-axis [113,118] or *y*-axis [112,119] can also be used.

Sensors for BCG are diverse, including bed-based EMFi film sensors [120,121], bed-based loadcell sensors [122], bed-based hydraulic sensors [123], scale-based sensors [124], PVDF sensors [125,126], etc. Accelerometer-based BCG measurements can be acquired by inserting a micro-electro-mechanical system (MEMS) accelerometer into a chair [118] or by attaching one to the center of mass (CoM) of the human body [127]. Unlike scale-based sensors, integrated sensors (e.g., hydraulic, EMFi, PVDF, etc.) and accelerometers can offer continuous BCG measurements. Furthermore, accelerometer-based BCG is wearable compared with bed-based sensors.

SCG and GCG measurements can be acquired by attaching an accelerometer or a gyroscope, respectively, to the sternum. A complete overview of the sensors that can be employed for SCG and GCG recordings is reported by Taebi et al. [128]. MEMS is a popular choice of accelerometer when the sensor is employed for HR extraction. For example, Tadi et al. [112,129] used a triaxial MEMS accelerometer to record SCG signals from the sternum of human subjects in resting state, where true positive beat detection rates of 95.8%, 99.3%, and 99.5% were achieved, respectively, for supine, left lateral, and right lateral positions. As an alternative to SCG, GCG measurements are preferably recorded using a MEMS gyroscope due to its size, cost, power consumption, and accuracy [115,130]. Moreover, MEMS gyroscopes measure angular velocity based on Coriolis force [119], which makes the collected GCG less affected by posture changes of the human subject as compared to SCG [116]. GCG alone can be used to extract the HR based on beat-to-beat detection as demonstrated by Wahlström et al. [131], with an absolute error of the beat-to-beat HR of 0.29±0.36 bpm. Gardner et al. [132] used a triaxial MEMS gyroscope embedded in a continuous positive airway pressure (CPAP) mask to measure GCG and achieved a beat-to-beat HR error of 2.35±2.30 bpm.

### 4.3. Signal Processing Approaches to Extract the HR

Using ECG as reference, the performances of different approaches from the literature for stand-alone beat-to-beat HR extraction of contact-based MCG measurements are shown in Table 2. As can be observed, despite the fact that different evaluation metrics have been employed, stand-alone beat-to-beat detection based on MCG measurements shows good performance.

Generally speaking, the approaches for extracting the HR from MCG measurements that are presented in the literature follow a similar strategy. First, respiration and motion artifacts are removed as a preprocessing stage. After preprocessing, the HR is estimated.

Unprocessed MCG measurements usually contain a respiratory component that can be suppressed by high-pass filtering [133]. Furthermore, due to the nature of the motion sensors, MCG measurements are in general more susceptible to random motion distortions than ECG measurements. Motion artifacts found in MCG measurements collected via an accelerometer or gyroscope (i.e., contact-based) are usually irregular and intermingled with heartbeat-induced motion in both the time and frequency domains [140,141]. This makes it challenging to estimate the HR, especially the beat-to-beat HR. In recent years, most research on MCG measurements has therefore focused on the effective removal of motion artifacts. Some studies suggest the use of two or more accelerometers and/or gyroscopes, with at least one sensor attached to the sternum and at least one to the right side of the back. With blind source separation techniques, motion artifacts can subsequently be effectively removed from sternal cardio-mechanical measurements [134,139]. However, unlike single-sensor approaches, the usage of multiple sensors increases the complexity of the measurement setup and the computational complexity of the processing stage.

To overcome such limitations, motion artifact removal based on single accelerometer measurements has also been investigated. Javaid et al. [142] employed an empirical mode decomposition (EMD) based approach to increase the SNR of the collected SCG measurements during walking. Yu et al. [133] proposed a novel adaptive recursive least squares (ARLS) filter to remove motion artifacts in SCG measurements that were collected during the standing and walking movements of the subjects. Motion artifacts found in bed-based [121] and scale-based [143] BCG measurements were also tackled, where the former detected motion artifacts based on dual thresholds, and the latter added secondary strain gauge sensors to the scale to detect motion artifacts.

As mentioned above, after denoising and artifact removal, the HR can be estimated. The algorithms that have been proposed in the literature for extracting the beat-to-beat HR from MCG measurements, independent of a reference (e.g., ECG), can be divided into three main categories: time-domain analysis, wavelet analysis, and artificial intelligence (AI) based approaches. For real-time applications, the employed approaches for beat-to-beat HR estimation rely on beat detection in the time domain analysis, including Hilbert transform [112], envelop-based detection [135], an autocorrelated differential algorithm (ADA) [113], and template matching methods [122]. For performance optimization, while real-time applications are not required, research is currently exploring methods such as wavelet analysis [136] and AI [139] to overcome non-stationary and irregular patterns that can be present in long-term MCG measurements.

### 4.4. Extramural Applications

Thanks to recent technological developments, reduced costs, and simplified implementation of the measurement setup (e.g., MEMS), there is increasing interest in MCG measurements for extramural monitoring purposes. Such extramural applications can be divided into those performed at home and those related to ambulant scenarios. For the first scenario, it is assumed that the user remains still during the recording, as this limits the presence of motion artifacts in the acquired signals. BCG/SCG measurements can be collected multiple times a day or continuously overnight. Inan et al. [124] used a modified commercially available scale to obtain repeatable BCG measurements with sufficient quality in terms of I-J amplitude and timing.

To limit obtrusiveness, sensors can be embedded in objects at home. For example, Cao et al. [144] used a chair with a load-cell platform installed under the seat to measure BCG. However, similar to the work by Inan et al. [124], this study relied on the R-peak detection of ECG to locate J and K waves of BCG, which rendered them unfeasible as a standalone BCG measurement setup for HR estimation. Lydon et al. [123] used a hydraulic bed sensor that can be placed under a mattress to collect BCG measurements from four elderly people in their apartments while they lay on their beds. They also proposed a short-time energy-based method to detect heartbeats in BCG measurements and the results seemed promising (Sensitivity/Specificity = 100%/95%) even with the presence of motion distortions. Regarding SCG, Ashouri et al. [145] proposed an algorithm that can automatically detect accelerometer misplacement, which can be potentially exploited for HR monitoring at home.

In ambulant settings, where human subjects perform daily activities (e.g., walking), the commonly used sensing modality is a wearable accelerometer for BCG/SCG acquisition. Rienzo et al. [146] used the MagIC-SCG garment with an accelerometer located in the vest pocket at the sternum level and tested the feasibility of continuously measuring the SCG signal in one human subject from 8 am to 8 pm and successfully estimated beat-to-beat cardiac time intervals using ECG as a reference. Despite promising results, data collection on more human subjects is needed to verify the usability of ambulatory BCG/SCG. Yang et al. [139] employed a dual-SCG system, which was proposed in [134] and is composed of two accelerometers, to monitor HR in 14 subjects who performed jogging and walking exercises. The relatively high detection accuracy (91.6% and 87.6% were achieved, respectively, for walking and jogging scenarios) suggests a high potential for using a wearable SCG for daily ambulatory HR monitoring. As for BCG measurement, Javaid et al. [147] employed a multi-sensor-fusion approach to record multi-site BCG measurements while the four human participants were performing a walking activity and successfully obtained systolic time intervals that demonstrated good correlation (r=0.7) with impedance cardiogram. However, more studies on the feasibility of using wearable BCG for HR estimation in ambulant subjects are still needed.

### 4.5. Future Developments

In addition to contact-based MCG sensors, research is also being carried out on contact-free sensing modalities for MCG measurements. Existing technologies that allow contact-free acquisition of MCG include radio frequency (RF) technology [148,149,150], laser Doppler vibrometry (LDV) [151,152], airborne ultrasound [153], and laser speckle vibrometry (SV). RF technology processes the phase variation information of a microwave radar signal to extract heartbeats [128], which is mainly BCG due to its delocalized area of interest (AOI). Unlike RF, airborne ultrasound can restrict its AOI and detect SCG [153]. LDV detects the vibration velocity of the surface where the laser spot is focused, by determining the frequency shift between the emitted and reflected laser beams [154]. In this case, the detected MCG measurement is essentially SCG. SV exploits the laser speckle effect, where a laser speckle pattern is formed on an optically rough surface and is extremely sensitive to temporal changes induced by both tiny linear acceleration and rotational motions on that surface [155]. Based on this principle, the MCG measurement extracted using SV is a composition of both GCG and SCG.

The success of RF technology in general is subject to the complexity of separating cardiac motions from respiratory ones, which results in difficulties in beat-to-beat HR extraction. Compared to RF, LDV has a more localized AOI, and thus better performance on beat-to-beat HR extraction. However, LDV is angle-sensitive in the sense that the laser beam should be perpendicular to the detected surface (e.g., the sternum). Moreover, the measured surface has to be reasonably reflective [128,156] which often requires the use of retro-reflective materials on the skin [157,158]. Their cost and size also pose limitations for usage and integration with other devices. Similar to RF, airborne ultrasound can detect heartbeat through textile [153]. The downside of using airborne ultrasound is the complexity in precise aiming (i.e., the selection of anatomical locations), which usually requires a trade-off in the distance of measurement or the implementation of multiple speakers [159]. SV can extract heartbeats with a laser on the chest area [160,161] while skin exposure is not necessarily needed. A SV-based contact-free monitor validation study was carried out and an average accuracy of more than 99% was achieved in beat-to-beat detection in 115 human subjects in a sitting position [162]. Unlike LDV, SV is not angle-sensitive and does not pose a strict requirement on the reflective properties of the measured surface. However, similarly to the three sensing modalities mentioned above, SV is susceptible to motion distortions.

The existing contact-free methods for MCG acquisition, including LDV and airborne ultrasound, are currently all still too cumbersome and expensive to be implemented at home for extramural monitoring purposes. A commercial SV-based HR monitor [162] seems to be a suitable candidate but requires validation. Furthermore, extensive investigations into the identification and mitigation of potential motion artifacts present in these contact-free sensing modalities are still lacking. To increase their potential in extramural monitoring, future studies need to look into system designs that allow these technologies to be integrated into a domestic environment.

## 5. Comparison of HR Estimation with the Different Sensing Modalities

In this section, we directly compare the solutions proposed within the three categories described above (i.e., sensing of electrical activity, peripheral activity of the heart, and mechanical activity). For each class, we discuss the most recent and advanced solutions proposed in the literature for extramural HR monitoring. We propose a comparison of several characteristics in order to help the readers in identifying which is the most suitable solution for their application, providing a guide for the selection of the appropriate technology.

Table 3 reports several criteria for each category. When possible, we propose a ranking from the best (rank = 1) to the worst (rank = 3) of the three sensing categories.

The evaluated characteristics can be divided into three main classes (i.e., general, hardware, and signal processing), which are discussed below.

### 5.1. General

The most important general characteristic that we discuss is the validation provided in the literature for the technologies considered. ECG-based HR monitoring has been widely and extensively investigated for decades in various scenarios, ranging from normal daily activity (e.g., during fitness training [55] or driving a car [42]) to challenging settings (e.g., underwater [163], climbing [164], etc.). This technology, by means of the Holter device, was the first portable option to monitor HR in extramural scenarios. Then, it was improved by testing and validating the performance by employing different types of electrodes and materials. For this reason, the American Heart Association [165] considers the ECG to be the gold standard for investigations of heart rhythm and HR monitoring. However, the high cost (about EUR 500 for QardioCore (Qardio^®^, San Francisco, CA, USA) and EUR 300 for Withings ScanWatch (Withings^®^, Paris, France)) of the commercially handled ECG devices makes them a high-end device unsuitable for wide-range commercial applications that are unrelated to medical purposes. Therefore, in recent years wearable devices based on PPG technology have been proposed, since they can be realized with cheaper hardware than ECG. In fact, the cost of the cheapest commercial PPG wristband is less than EUR 20, making it affordable for the majority of the population. The high demand for PPG-based HR monitors from users who want to check their physiological parameters during normal daily activities or fitness training has led to extensive research development and validation of this device in recent years. This increased interest from research and commerce led to an improvement in accuracy in HR estimation and also in challenging scenarios, such as during intense physical exercise that involves poor signal quality [76]. HR monitoring based on the mechanical activity of the heart is the youngest technique among those described in this paper. Therefore, the validation of MCG and its performance are still under investigation by the scientific community. Furthermore, one of the main obstacles to its commercial spread and wide application is related to the lack of standard measurement methodologies and protocols [109]. Since the application and validation of inertial sensors to measure the mechanical activity of the heart are still in a preliminary phase, MCG-based devices are not yet available on the market. However, accelerometers/gyroscopes are usually cheap (around EUR 10), so when the research in this field will be able to provide commercial devices (for both medical and non-medical applications), it is expected that their cost will not be high.

Finally, the last general item we considered to compare the three sensor modalities described in this paper is the availability of contact-free measurement conditions, which means that no sensors are placed on the body to provide the HR estimation. ECG sensors cannot reach this possibility, since they are sensitive to the electrical variation induced by heart activity only if they are placed on the body. In contrast, PPG technology can be used remotely via a camera that can detect skin color variations due to pulsatile blood flow in the most superficial capillaries [166]. Furthermore, for MCG sensors the radio frequency and ultrasound technologies that are used to detect the vibrating activity of the body induced by heart pumping can also be placed relatively far from the body [128,153]. Contact-free measurements have the undoubted advantage of minimizing intrusiveness; however, providing the right exposure of the body to the camera or the ultrasound probe is quite difficult and loss of signal is frequent. Furthermore, the portability of such contact-free instrumentation is limited because the measurement setup cannot easily follow the user in outdoor scenarios. Therefore, its application is still limited to a few convenient scenarios, such as the HR monitoring of car drivers [52]. For all these reasons, beyond the great potential of this modality, further investigations are needed in the future.

### 5.2. Hardware

The first item evaluated for the hardware is the dimension of the sensors, as reported in Table 3. As discussed in Section 2.1, the ECG signal can only be measured as the potential difference between two points of the body surface. Therefore, at least two electrodes are needed, and a third ground electrode is commonly used to improve SNR. Furthermore, if the electrodes are placed too close to each other, the acquired signal will have a low amplitude, which will make the R-peaks detection challenging, causing poor HR estimation. Therefore, portable ECGs are more suitable for being embedded in objects with a large surface (e.g., belt, t-shirt, bed, chairs, and so on), rather than in a smartwatch or fitness band. The optical measurement performed by the PPG devices requires two elements (i.e., the led that transmits the light and the photodetector that receives it). However, unlike the ECG, these two elements need to be close to each other to avoid dispersion of light, which would reduce the acquired signal. For these reasons, such sensors are suitable for compact devices, such as smartwatches or smart bands. Finally, contact-based MCG sensors rely on an accelerometer or a gyroscope. Therefore, since only one probe is needed, this solution is the one that involves a smaller size than the other solutions considered in this paper.

The second hardware criterion that we consider is skin exposure. For PPG sensors, skin exposure is a mandatory requirement because the optical measurement would be hampered by the presence of a layer of material between the skin and the sensors. The ECG is sensitive to the electrical field of the body, and the MCG to the vibration induced by heart activity. Therefore, the presence of a layer between the skin and the electrodes (e.g., a fabric) does not make the detection unfeasible, but often reduces the SNR.

Finally, we discuss the robustness of the considered modalities to motion artifacts, which is a major challenge for HR monitors employed during extramural activities. Traditional wet ECG electrodes are not very sensitive to movements; therefore, ECG acquisitions can have high quality also in presence of motion. On the other hand, the signals acquired with capacitive electrodes usually have lower quality because they are affected by motion artifacts induced by changes in the distance between the electrodes and the skin. The PPG sensors need to be positioned in parts of the body where the capillaries are superficial (e.g., wrist, fingers, earlobes, etc.), to obtain a strong and reliable signal. However, the downside of these positions is that they are located in parts of the body that are subject to frequent movements even during normal daily activities and which are intensified during physical activity. Therefore, signals acquired with PPG sensors, whose quality is highly sensitive to motion, are often corrupted by a large number of motion artifacts that make the accurate and continuous detection of HR difficult. MCG sensors are based on the detection of micro-motions induced by the heart pumping; therefore, they are in general more susceptible to motion distortions than ECG sensors. For these types of sensors as well as for PPG sensors, the employment of additional sensors, which help in the removal of the artifacts, is a strategy often pursued [79,167,168,169].

### 5.3. Signal Processing

The last category that we consider for the comparison is signal processing. As discussed in the previous sections, the extraction of a reliable HR estimation in extramural scenarios could be a challenging task due to low SNR and motion artifacts that corrupt the signals. Each sensing modality has different abilities in HR detection and it is important to take its shortcomings and strengths into account in the selection and design phase of the monitoring approach.

The waveforms of the signals acquired with the sensors discussed in this article are very different from each other. The ECG signal is characterized by the high amplitude of the R waves and limited motion artifacts, thanks to favorable sensor placement, in locations in the body that are less subject to movement than others. This makes the detection of R peaks in the time domain feasible by using simple and computationally light methods based on adaptive thresholds. In this way, an accurate beat-to-beat HR estimation is provided. If the acquisitions are made under non-optimal conditions with noise and artifacts that corrupt the signal, the threshold detector is often preceded by a denoising phase, as discussed in Section 2.3. These strategies have low computational complexity, requiring limited processing time, and can facilitate that the HR estimation can be provided to the user in real-time and with high accuracy. For example, Lazaro et al. [51] estimate the HR from ECG signals acquired via dry electrodes embedded in an armband while the user performs normal daily activities with an accuracy of 98.54%. The reference in their study is ECG signals acquired by means of a Holter device.

In Section 3.3, we already discussed the sensitivity of PPG signals to artifacts that on the one hand are caused by the positioning of optical sensors on body parts that are frequently moved (e.g., wrist, finger), and on the other hand by the measurement principle of the PPG which makes is, in comparison to ECG, extra sensitive to motion-induced distortions of the PPG wave shape, even when the motion has low intensity. Due to this high sensitivity, the extraction of HR in an extramural monitoring scenario is challenging. To overcome this limitation, PPG devices are often equipped with additional inertial sensors (e.g., accelerometers) that provide information on artifacts to make denoising more effective. The denoising approaches proposed in the literature are abundant and based on different strategies, such as signal decomposition [50,170], Wiener filtering [171], or supervised learning frameworks [172]. Some of those are computationally complex; however, thanks to the high computational ability of the recently proposed smartphones or smartwatches, this shortcoming can be handled. Furthermore, to improve reliability, the HR estimation is usually performed in the frequency domain, which is more suitable for rejecting artifacts than the time domain estimation. Such an approach provides a highly accurate estimation (error lower than 1 bpm [79]) but cannot perform a beat-to-beat estimation, being unsuitable for some types of HRV analysis. Very few studies presented in the literature attempt to overcome these limitations, suggesting that research in this field is still in an emerging phase. For instance, Jarchi et al. [173] investigated a processing method based on instantaneous spectral measures that were calculated using empirical mode decomposition and Hilbert Transform with a novel spectral masking stage. Successful estimation of HR (the mean error is equal to 1.75 bpm) provides a clue that a beat-to-beat HR estimation could be extensively used in the future after further investigations.

To be effectively adopted in extramural monitoring scenarios, MCG measurements require the development of more advanced processing strategies than those already presented in the literature. In fact, due to the high amount and variability of artifacts induced by movements, it is still relatively far from possible to provide an accurate beat-to-beat estimate of HR outside laboratories and clinical settings. One of the most pioneering studies presented on this topic [139] uses an array of inertial sensors (accelerometers for SCG or gyroscopes for GCG) combined with a processing strategy based on independent component analysis. This approach allows obtaining an acceptable beat-to-beat HR estimation during jogging (Se = 86.06% for SCG and Se = 76.30% for GCG) and walking (Se = 91.44% for SCG and Se = 87.32% for GCG). However, this performance is lower than that can be obtained with ECG and PPG, and the processing algorithm is computationally expensive and could be unsuitable to be implemented in portable devices without introducing a delay in the HR estimation.

Through beat-to-beat HR estimation, HRV can be calculated and used to detect arrhythmias. The ECG signal is considered by the medical and scientific community the gold standard for the diagnosis and detection of heart rhythm-related pathologies, thanks to the high level of accuracy achieved in detection. For example, Marsili et al. [174] achieved 98.1% accuracy in real-time detection of atrial fibrillation (AF) through their algorithm implemented onboard a wearable ECG device. The state-of-the-art PPG and MCG-based methods also show good accuracy for AF detection. For instance, regarding PPG-based sensors, Solosenko et al. [175] and Yang et al. [176] obtained an accuracy of 87% and 92.71%, respectively. Instead, Hurnanen et al. [177] who employed SCG measurements achieved a True Positive Rate of 99.9% and a True Negative Rate of 96.4% in the classification of AF segments. However, these studies were carried out in hospital settings. Therefore, further investigation is required for the validation of PPG and MCG sensors for the detection of atrial fibrillation in extramural and ambulant scenarios [178].

## 6. Conclusions

This work provides an overview of the sensing strategies capable of detecting the three main correlates of cardiac function. In particular, we focus on the electrical activity of the heart, detected with the electrocardiogram, the peripheral effects of the heart activity, recorded with the photoplethysmogram, and the mechanical activity of the heart, represented with the mechanocardiogram. For each of these sensing modalities, we illustrate the physical principle used for the measurement, the sensors employed for the detection, and the signal processing approaches proposed to extract HR from the acquired signals. Furthermore, we discuss for each of the three modalities which are their possible extramural applications and future developments. Finally, Section 5 proposes a comprehensive comparison of different aspects (i.e., general, hardware, and signal processing) of the considered sensing modalities. This conclusive section aims at helping the readers to identify which is the most suitable solution for their application, providing helpful guidelines for appropriate technology selection.

## Figures and Tables

**Table 1:** Summary of instrumentation for mechanocardiogram (MCG).

MCG Measurements	Measurement Origin	Contact-Based Sensing Modalities	Contact-Free Sensing Modalities
**BCG**	Whole-body recoil movement	Scale, Hydraulic sensors, EMFi film sensors, Accelerometer, etc.	Radio Frequency
**SCG**	Sternal accelerations	Accelerometer	Laser Doppler Vibrometer, Laser Speckle Vibrometry, Airborne Ultrasound
**GCG**	Sternal rotations	Gyroscope	Laser Speckle Vibrometry

**Table 2:** Performances on beat-to-beat heart rate estimation of MCG using ECG as reference. Se: sensitivity; Sp: specificity; Acc: accuracy; Err = error.

Algorithms	Measurement	# Subjects	Body Posture	Beat-to-Beat Detection Performances
Time domain analysis	SCG SCG SCG SCG SCG + GCG BCG BCG BCG	16 10 29 20 25 33 3 10	walking walking supine sitting supine supine supine supine	Acc = 98% [133] Se = 98.7% [134] Se/Sp = 99.5%/99.8% [112] Acc = 98.3% [135] Se/Sp = 96.6%/99.7% [113] Err = 0.83% [120] Se/Sp = 84%/90% [121] Se/Sp = 95.2%/94.8% [122]
Wavelet analysis	SCG	17	supine	Err = 2.27 ± 0.81 bpm [136]
Aritifical intelligence	BCG SCG SCG + GCG SCG GCG	17 20 67 14 14	supine supine supine walking/jogging walking/jogging	Se/Sp = 76%/85% [137] Se/Sp = 98.5%/98.6% [138] Err = 0.56 ± 2.74 bpm [131] Se = 91.6% ± 2.1/86.4% ± 4.1 [139] Se = 87.6% ± 3.9/76.8% ± 5.5 [139]

**Table 3:** Comparison between the three sensing categories (i.e., sensing of electrical activity, peripheral activity of the heart and mechanical activity). The considered criteria are divided in three classes (i.e., general, hardware and signal processing). When possible we introduced a ranking from best (rank = 1) to worst (rank = 3) of the sensing modalities.

		Electrical	Peripheral	Mechanical
		Activity-Based	Effect-Based	Activity-Based
**General**	validated	1	2	3
	low-cost equipment	2	1	(*)
	contact-free	no	yes	yes
**Hardware**	dimension	3	2	1
	skin exposure	1	2	1
	motion artifacts robustness	1	3	2
**Signal processing**	light-weight processing	1	2	2
	HR estimation accuracy	1	2	3
	real time estimation	yes	yes	yes
	anomalous rhythm detection	yes	yes	yes

(*) No commercial devices are still available in the market for this type of sensors.

## References

[1] Venes D. (2017). Taber’s Cyclopedic Medical Dictionary.

[2] Rajendra Acharya U., Paul Joseph K., Kannathal N., Lim C.M., Suri J.S. (2006). Heart rate variability: A review. Med. Biol. Eng. Comput..

[3] Betts J.G., Young K.A., Wise J.A., Johnson E., Poe B., Kruse D.H., Korol O., Johnson J.E., Womble M., DeSaix P. (2013). Anatomy and Physiology.

[4] Saeed M., Villarroel M., Reisner A.T., Clifford G., Lehman L.W., Moody G., Heldt T., Kyaw T.H., Moody B., Mark R.G. (2011). Multiparameter Intelligent Monitoring in Intensive Care II (MIMIC-II): A public-access intensive care unit database. Crit. Care Med..

[5] Fidler R.L., Pelter M.M., Drew B.J., Palacios J.A., Bai Y., Stannard D., Aldrich J.M., Hu X. (2017). Understanding heart rate alarm adjustment in the intensive care units through an analytical approach. PLoS ONE.

[6] Palatini P. (1999). Need for a revision of the normal limits of resting heart rate. Hypertension.

[7] Shaffer F., Ginsberg J.P. (2017). An overview of heart rate variability metrics and norms. Front. Public Health.

[8] Kovatchev B.P., Farhy L.S., Cao H., Griffin M.P., Lake D.E., Moorman J.R. (2003). Sample asymmetry analysis of heart rate characteristics with application to neonatal sepsis and systemic inflammatory response syndrome. Pediatr. Res..

[9] Hasty F., García G., Dávila H., Wittels S.H., Hendricks S., Chong S. (2021). Heart rate variability as a possible predictive marker for acute inflammatory response in COVID-19 patients. Mil. Med..

[10] Benedetto S., Caldato C., Greenwood D.C., Bartoli N., Pensabene V., Actis P. (2019). Remote heart rate monitoring-Assessment of the Facereader rPPg by Noldus. PLoS ONE.

[11] Dong J.G. (2016). The role of heart rate variability in sports physiology. Exp. Ther. Med..

[12] Carter R., Cheuvront S.N., Wray D.W., Kolka M.A., Stephenson L.A., Sawka M.N. (2005). The influence of hydration status on heart rate variability after exercise heat stress. J. Therm. Biol..

[13] Kudaiberdieva G., Görenek B., Timuralp B. (2007). Heart rate variability as a predictor of sudden cardiac death. Anatol. J. Cardiol..

[14] Papini G.B., Fonseca P., van Gilst M.M., van Dijk J.P., Pevernagie D.A., Bergmans J.W., Vullings R., Overeem S. (2019). Estimation of the apnea-hypopnea index in a heterogeneous sleep-disordered population using optimised cardiovascular features. Sci. Rep..

[15] Hernando D., Roca S., Sancho J., Alesanco Á., Bailón R. (2018). Validation of the apple watch for heart rate variability measurements during relax and mental stress in healthy subjects. Sensors.

[16] Roberts D.M., Schade M.M., Mathew G.M., Gartenberg D., Buxton O.M. (2020). Detecting sleep using heart rate and motion data from multisensor consumer-grade wearables, relative to wrist actigraphy and polysomnography. Sleep.

[17] Tirziu D., Giordano F.J., Simons M. (2010). Cell communications in the heart. Circulation.

[18] Barold S.S. (2003). Willem Einthoven and the birth of clinical electrocardiography a hundred years ago. Card. Electrophysiol. Rev..

[19] Li K.H.C., White F.A., Tipoe T., Liu T., Wong M.C., Jesuthasan A., Baranchuk A., Tse G., Yan B.P. (2019). The current state of mobile phone apps for monitoring heart rate, heart rate variability, and atrial fibrillation: Narrative review. JMIR mHealth uHealth.

[20] Tu H.T., Chen Z., Swift C., Churilov L., Guo R., Liu X., Jannes J., Mok V., Freedman B., Davis S.M. (2017). Smartphone electrographic monitoring for atrial fibrillation in acute ischemic stroke and transient ischemic attack. Int. J. Stroke.

[21] Iskandar A.A., Kolla R., Schilling K., Voelker W. A wearable 1-lead necklace ECG for continuous heart rate monitoring. Proceedings of the 2016 IEEE 18th International Conference on e-Health Networking, Applications and Services (Healthcom).

[22] Chi Y.M., Jung T.P., Cauwenberghs G. (2010). Dry-Contact and Noncontact Biopotential Electrodes: Methodological Review. IEEE Rev. Biomed. Eng..

[23] Gruetzmann A., Hansen S., Müller J. (2007). Novel dry electrodes for ECG monitoring. Physiol. Meas..

[24] Ramasamy S., Balan A. (2018). Wearable sensors for ECG measurement: A review. Sens. Rev..

[25] Fu Y., Zhao J., Dong Y., Wang X. (2020). Dry electrodes for human bioelectrical signal monitoring. Sensors.

[26] Guzik P., Malik M. (2016). ECG by mobile technologies. J. Electrocardiol..

[27] Galli A., Peri E., Zhang Y., Vullings R., van der Ven M., Giorgi G., Ouzounov S., Harpe P.J., Mischi M. (2021). Dedicated algorithm for unobtrusive fetal heart rate monitoring using multiple dry electrodes. Sensors.

[28] Meziane N., Webster J., Attari M., Nimunkar A. (2013). Dry electrodes for electrocardiography. Physiol. Meas..

[29] Chlaihawi A.A., Narakathu B.B., Emamian S., Bazuin B.J., Atashbar M.Z. (2018). Development of printed and flexible dry ECG electrodes. Sens. Bio-Sens. Res..

[30] Niu X., Gao X., Liu Y., Liu H. (2021). Surface bioelectric dry Electrodes: A review. Measurement.

[31] Zheng K., Chen S., Zhu L., Zhao J., Guo X. (2018). Large area solution processed poly (dimethylsiloxane)-based thin film sensor patch for wearable electrocardiogram detection. IEEE Electron Device Lett..

[32] Kim T., Park J., Sohn J., Cho D., Jeon S. (2016). Bioinspired, highly stretchable, and conductive dry adhesives based on 1D–2D hybrid carbon nanocomposites for all-in-one ECG electrodes. ACS Nano.

[33] Castrillón R., Pérez J.J., Andrade-Caicedo H. (2018). Electrical performance of PEDOT: PSS-based textile electrodes for wearable ECG monitoring: A comparative study. Biomed. Eng. Online.

[34] Pani D., Dessì A., Saenz-Cogollo J.F., Barabino G., Fraboni B., Bonfiglio A. (2015). Fully textile, PEDOT: PSS based electrodes for wearable ECG monitoring systems. IEEE Trans. Biomed. Eng..

[35] Bihar E., Roberts T., Saadaoui M., Hervé T., De Graaf J.B., Malliaras G.G. (2017). Inkjet-printed PEDOT: PSS electrodes on paper for electrocardiography. Adv. Healthc. Mater..

[36] Sinha S.K., Noh Y., Reljin N., Treich G.M., Hajeb-Mohammadalipour S., Guo Y., Chon K.H., Sotzing G.A. (2017). Screen-printed PEDOT: PSS electrodes on commercial finished textiles for electrocardiography. ACS Appl. Mater. Interfaces.

[37] Nigusse A.B., Mengistie D.A., Malengier B., Tseghai G.B., Langenhove L.V. (2021). Wearable Smart Textiles for Long-Term Electrocardiography Monitoring—A Review. Sensors.

[38] Nemati E., Deen M.J., Mondal T. (2012). A wireless wearable ECG sensor for long-term applications. IEEE Commun. Mag..

[39] Ryu C.Y., Nam S.H., Kim S. Conductive rubber electrode for wearable health monitoring. Proceedings of the 2005 IEEE Engineering in Medicine and Biology 27th Annual Conference.

[40] Lim Y.G., Kim K.K., Park S. (2006). ECG measurement on a chair without conductive contact. IEEE Trans. Biomed. Eng..

[41] Baek H.J., Chung G.S., Kim K.K., Park K.S. (2011). A smart health monitoring chair for nonintrusive measurement of biological signals. IEEE Trans. Inf. Technol. Biomed..

[42] Uguz D.U., Dettori R., Napp A., Walter M., Marx N., Leonhardt S., Hoog Antink C. (2020). Car seats with capacitive ECG electrodes can detect cardiac pacemaker spikes. Sensors.

[43] Varadan V.K., Kumar P.S., Oh S., Kegley L., Rai P. (2011). e-bra With Nanosensors for Real Time Cardiac Health Monitoring and Smartphone Communication. J. Nanotechnol. Eng. Med..

[44] Biagetti G., Crippa P., Falaschetti L., Orcioni S., Turchetti C. (2016). Wireless surface electromyograph and electrocardiograph system on 802.15. 4. IEEE Trans. Consum. Electron..

[45] Galli A., Giorgi G., Narduzzi C. Multi-user ECG monitoring system based on IEEE standard 802.15. 6. Proceedings of the 2019 IEEE International Symposium on Measurements & Networking (M&N).

[46] Pan J., Tompkins W.J. (1985). A real-time QRS detection algorithm. IEEE Trans. Biomed. Eng..

[47] Galli A., Frigo G., Giorgi G. Robust ECG Denoising for eHealth applications. Proceedings of the 2018 IEEE International Symposium on Medical Measurements and Applications (MeMeA).

[48] Peng S., Bao S., Chen W. Capacitive coupled electrodes based non-contact ECG measurement system with real-time wavelet denoising algorithm. Proceedings of the 2019 41st Annual International Conference of the IEEE Engineering in Medicine and Biology Society (EMBC).

[49] Kota D., Tasneem N., Kakaraparty K., Mahbub I., Mehta G., Namuduri K. (2021). A Low-power Dry Electrode-based ECG Signal Acquisition with De-noising and Feature Extraction. J. Signal Process. Syst..

[50] Galli A., Frigo G., Chindamo D., Depari A., Gadola M., Giorgi G. (2019). Denoising ECG signal by CSTFM algorithm: Monitoring during motorbike and car races. IEEE Trans. Instrum. Meas..

[51] Lázaro J., Reljin N., Hossain M.B., Noh Y., Laguna P., Chon K.H. (2020). Wearable armband device for daily life electrocardiogram monitoring. IEEE Trans. Biomed. Eng..

[52] Wu T., Redouté J.M., Yuce M. (2019). A wearable, low-power, real-time ECG monitor for smart t-shirt and IoT healthcare applications. Advances in Body Area Networks I.

[53] Chamadiya B., Mankodiya K., Wagner M., Hofmann U.G. (2013). Textile-based, contactless ECG monitoring for non-ICU clinical settings. J. Ambient. Intell. Humaniz. Comput..

[54] Eilebrecht B., Czaplik M., Walter M., Wartzek T., Rossaint R., Leonhardt S. (2009). Implementation of a capacitive ECG measurement system in clinical practice: An interim report. Proceedings of the World Congress on Medical Physics and Biomedical Engineering.

[55] Patel R.K., Gupta A., Chowdary V., Kaundal V., Mondal A.K. (2019). Wearable fitness band-based U-health monitoring. Sensors for Health Monitoring.

[56] Worringham C., Rojek A., Stewart I. (2011). Development and feasibility of a smartphone, ECG and GPS based system for remotely monitoring exercise in cardiac rehabilitation. PLoS ONE.

[57] Baig M.M., Gholamhosseini H., Connolly M.J. (2013). A comprehensive survey of wearable and wireless ECG monitoring systems for older adults. Med. Biol. Eng. Comput..

[58] El Attaoui A., Hazmi M., Jilbab A., Bourouhou A. (2020). Wearable wireless sensors network for ECG telemonitoring using neural network for features extraction. Wirel. Pers. Commun..

[59] Schuchert A., Behrens G., Meinertz T. (1999). Impact of long-term ECG recording on the detection of paroxysmal atrial fibrillation in patients after an acute ischemic stroke. Pacing Clin. Electrophysiol..

[60] Selvaraj N. Assessment of pulse transit/arrival time as noninvasive blood pressure predictors in finger and earlobe sites. Proceedings of the 2016 IEEE Healthcare Innovation Point-Of-Care Technologies Conference (HI-POCT).

[61] Allen J. (2007). Photoplethysmography and its application in clinical physiological measurement. Physiol. Meas..

[62] Elgendi M. (2012). On the Analysis of Fingertip Photoplethysmogram Signals. Curr. Cardiol. Rev..

[63] Abay T.Y., Kyriacou P.A. (2015). Reflectance Photoplethysmography as Noninvasive Monitoring of Tissue Blood Perfusion. IEEE Trans. Biomed. Eng..

[64] Tamura T., Maeda Y., Sekine M., Yoshida M. (2014). Wearable Photoplethysmographic Sensors—Past and Present. Electronics.

[65] Gajinov Z., Matić M., Prćić S., Đuran V. (2013). Optical properties of the human skin/Optičke osobine ljudske kože. Serbian J. Dermatol. Venereol..

[66] Kao Y.H., Chao P.C.P., Hung Y., Wey C.L. A new reflective PPG LED-PD sensor module for cuffless blood pressure measurement at wrist artery. Proceedings of the 2017 IEEE Sensors.

[67] Lee J., Matsumura K., Yamakoshi K.i., Rolfe P., Tanaka S., Yamakoshi T. Comparison between red, green and blue light reflection photoplethysmography for heart rate monitoring during motion. Proceedings of the 2013 35th Annual International Conference of the IEEE Engineering in Medicine and Biology Society (EMBC).

[68] Kamshilin A.A., Margaryants N.B. (2017). Origin of Photoplethysmographic Waveform at Green Light. Phys. Procedia.

[69] Brumfield A.M., Andrew M.E. (2005). Digital pulse contour analysis: Investigating age-dependent indices of arterial compliance. Physiol. Meas..

[70] Balmer J., Smith R., Pretty C.G., Desaive T., Chase J.G. (2021). Accurate end systole detection in dicrotic notch-less arterial pressure waveforms. J. Clin. Monit. Comput..

[71] Reguig F.B. Photoplethysmogram signal analysis for detecting vital physiological parameters: An evaluating study. Proceedings of the 2016 International Symposium on Signal, Image, Video and Communications (ISIVC).

[72] Shelley K.H., Alian A.A. (2014). Photoplethysmography. Best Pract. Res. Clin. Anaesthesiol..

[73] Leonhardt S., Leicht L., Teichmann D. (2018). A review on wearable photoplethysmography sensors and their potential future applications in health care. Int. J. Biosens. Bioelectron..

[74] Béres S., Hejjel L. (2021). The minimal sampling frequency of the photoplethysmogram for accurate pulse rate variability parameters in healthy volunteers. Biomed. Signal Process. Control..

[75] Maeda Y., Sekine M., Tamura T., Moriya A., Suzuki T., Kameyama K. Comparison of reflected green light and infrared photoplethysmography. Proceedings of the 2008 30th Annual International Conference of the IEEE Engineering in Medicine and Biology Society.

[76] Galli A., Narduzzi C., Giorgi G. (2017). Measuring heart rate during physical exercise by subspace decomposition and Kalman smoothing. IEEE Trans. Instrum. Meas..

[77] Allen J., Murray A. (2004). Effects of filtering on multisite photoplethysmography pulse waveform characteristics. Proceedings of the Computers in Cardiology.

[78] Chatterjee A., Roy U.K. PPG Based Heart Rate Algorithm Improvement with Butterworth IIR Filter and Savitzky-Golay FIR Filter. Proceedings of the 2018 2nd International Conference on Electronics, Materials Engineering Nano-Technology (IEMENTech).

[79] Biswas D., Simões-Capela N., Van Hoof C., Van Helleputte N. (2019). Heart Rate Estimation From Wrist-Worn Photoplethysmography: A Review. IEEE Sensors J..

[80] Wijshoff R.W., Mischi M., Veen J., Van Der Lee A.M., Aarts R.M. (2012). Reducing motion artifacts in photoplethysmograms by using relative sensor motion: Phantom study. J. Biomed. Opt..

[81] Papini G.B., Fonseca P., Aubert X.L., Overeem S., Bergmans J.W., Vullings R. Photoplethysmography beat detection and pulse morphology quality assessment for signal reliability estimation. Proceedings of the 2017 39th Annual International Conference of the IEEE Engineering in Medicine and Biology Society (EMBC).

[82] Goh C.H., Tan L., Lovell N., Ng S.C., Tan M., Lim E. (2020). Robust PPG motion artifact detection using a 1-D convolution neural network. Comput. Methods Programs Biomed..

[83] Pollreisz D., TaheriNejad N. (2019). Detection and Removal of Motion Artifacts in PPG Signals. Mob. Netw. Appl..

[84] Lai P.H., Kim I. (2015). Lightweight wrist photoplethysmography for heavy exercise: Motion robust heart rate monitoring algorithm. Healthc. Technol. Lett..

[85] Hara S., Shimazaki T., Okuhata H., Nakamura H., Kawabata T., Cai K., Takubo T. Parameter optimization of motion artifact canceling PPG-based heart rate sensor by means of cross validation. Proceedings of the 2017 11th International Symposium on Medical Information and Communication Technology (ISMICT).

[86] Liu J., Zhao Y., Lai B., Wang H., Tsui K.L. (2020). Wearable Device Heart Rate and Activity Data in an Unsupervised Approach to Personalized Sleep Monitoring. JMIR mHealth uHealth.

[87] Eerikäinen L., Bonomi A., Schipper F., Dekker L., Vullings R., de Morree H., Aarts R. How Accurately Can We Detect Atrial Fibrillation Using Photoplethysmography Data Measured in Daily Life? In Proceedings of the 2019 Computing in Cardiology (CinC), Singapore, 8–11 September 2019.

[88] Phan D., Siong L., Pathirana P., Seneviratne A. Smartwatch: Performance evaluation for long-term heart rate monitoring. Proceedings of the 2015 International Symposium on Bioelectronics and Bioinformatics (ISBB).

[89] Haque C.A., Hossain S., Kwon T.H., Kim K.D. Comparison of Different Methods to Estimate Blood Oxygen Saturation using PPG. Proceedings of the 2021 International Conference on Information and Communication Technology Convergence (ICTC).

[90] Evans M., Geddes L. (1988). An assessment of blood vessel vasoactivity using photoplethysmography. Med Instrum..

[91] Jönsson B., Laurent C., Eneling M., Skau T., Lindberg L.G. (2005). Automatic Ankle Pressure Measurements Using PPG in Ankle-brachial Pressure Index Determination. Eur. J. Vasc. Endovasc. Surg..

[92] Mansi S.A., Barone G., Forzano C., Pigliautile I., Ferrara M., Pisello A.L., Arnesano M. (2021). Measuring human physiological indices for thermal comfort assessment through wearable devices: A review. Measurement.

[93] McCombie D., Asada H., Reisner A. Identification of vascular dynamics and estimation of the cardiac output waveform from wearable PPG sensors. Proceedings of the 2005 IEEE Engineering in Medicine and Biology 27th Annual Conference.

[94] Barvik D., Cerny M., Penhaker M., Noury N. (2022). Noninvasive Continuous Blood Pressure Estimation From Pulse Transit Time: A Review of the Calibration Models. IEEE Rev. Biomed. Eng..

[95] Parak J., Korhonen I. Evaluation of wearable consumer heart rate monitors based on photopletysmography. Proceedings of the 2014 36th Annual International Conference of the IEEE Engineering in Medicine and Biology Society.

[96] Verkruysse W., Svaasand L.O., Nelson J.S. (2008). Remote plethysmographic imaging using ambient light. Opt. Express.

[97] Moço A., Verkruysse W. (2021). Pulse oximetry based on photoplethysmography imaging with red and green light. J. Clin. Monit. Comput..

[98] Zhang Y., Tsujikawa M., Onishi Y. Sleep/wake classification via remote PPG signals. Proceedings of the 2019 41st Annual International Conference of the IEEE Engineering in Medicine and Biology Society (EMBC).

[99] Sun Y., Hu S., Azorin-Peris V., Greenwald S., Chamers J., Zhu Y. (2011). Motion-compensated noncontact imaging photoplethysmography to monitor cardiorespiratory status during exercise. J. Biomed. Opt..

[100] McDuff D., Gontarek S., Picard R.W. (2014). Remote Detection of Photoplethysmographic Systolic and Diastolic Peaks Using a Digital Camera. IEEE Trans. Bio-Med. Eng..

[101] Gordon J. (1877). Certain molar movements of the human body produced by the circulation of the blood. J. Anat. Physiol..

[102] Salerno D.M., Zanetti J. (1991). Seismocardiography for monitoring changes in left ventricular function during ischemia. Chest.

[103] Starr I., Wood F.C. (1943). Studies with the ballistocardiograph in acute cardiac infarction and chronic angina pectoris. Am. Heart J..

[104] Giovangrandi L., Inan O.T., Wiard R.M., Etemadi M., Kovacs G.T. Ballistocardiography—A method worth revisiting. Proceedings of the 2011 Annual International Conference of the IEEE Engineering in Medicine and Biology Society.

[105] Scarborough W.R., Folk E.F., Smith P.M., Condon J.H. (1958). The nature of records from ultra-low frequency ballistocardiographic systems and their relation to circulatory events. Am. J. Cardiol..

[106] Starr I., Noordergraaf A. (1967). Ballistocardiography in Cardiovascular Research: Physical Aspects of the Circulation in Health and Disease.

[107] Starr I., Wood F.C. (1961). Twenty-year studies with the ballistocardiograph: The relation between the amplitude of the first record of “healthy” adults and eventual mortality and morbidity from heart disease. Circulation.

[108] Kim C.S., Ober S.L., McMurtry M.S., Finegan B.A., Inan O.T., Mukkamala R., Hahn J.O. (2016). Ballistocardiogram: Mechanism and potential for unobtrusive cardiovascular health monitoring. Sci. Rep..

[109] Guidoboni G., Sala L., Enayati M., Sacco R., Szopos M., Keller J.M., Popescu M., Despins L., Huxley V.H., Skubic M. (2019). Cardiovascular function and ballistocardiogram: A relationship interpreted via mathematical modeling. IEEE Trans. Biomed. Eng..

[110] Mounsey P. (1957). Praecordial ballistocardiography. Br. Heart J..

[111] Baevskii R.M., Egorov A.D., Kazarian L.A. (1964). Seismocardiography. Kardiologiia.

[112] Tadi M.J., Lehtonen E., Hurnanen T., Koskinen J., Eriksson J., Pänkäälä M., Teräs M., Koivisto T. (2016). A real-time approach for heart rate monitoring using a Hilbert transform in seismocardiograms. Physiol. Meas..

[113] D’Mello Y., Skoric J., Xu S., Roche P.J., Lortie M., Gagnon S., Plant D.V. (2019). Real-time cardiac beat detection and heart rate monitoring from combined seismocardiography and gyrocardiography. Sensors.

[114] Migeotte P.F., Lejeune L., Delière Q., Caiani E., Casellato C., Tank J., Funtova I., Baevsky R., Prisk G.K., van de Borne P. Three dimensional ballistocardiogram and seismocardiogram: What do they have in common?. Proceedings of the 2014 36th Annual International Conference of the IEEE Engineering in Medicine and Biology Society.

[115] Jafari Tadi M., Lehtonen E., Saraste A., Tuominen J., Koskinen J., Teräs M., Airaksinen J., Pänkäälä M., Koivisto T. (2017). Gyrocardiography: A new non-invasive monitoring method for the assessment of cardiac mechanics and the estimation of hemodynamic variables. Sci. Rep..

[116] MeriheinÄ U., Juppo M., Koivisto T., Pänkäälä M., Sairanen K., Grönholm M. (2015). Heart Monitoring System. Patent WO.

[117] Migeotte P.F., Mucci V., Delière Q., Lejeune L., Borne P.v.d. (2016). Multi-dimensional kineticardiography a new approach for wearable cardiac monitoring through body acceleration recordings. Proceedings of the XIV Mediterranean Conference on Medical and Biological Engineering and Computing 2016.

[118] Pinheiro E., Postolache O., Girão P. (2012). Study on ballistocardiogram acquisition in a moving wheelchair with embedded sensors. Metrol. Meas. Syst..

[119] Sieciński S., Kostka P.S., Tkacz E.J. (2020). Gyrocardiography: A review of the definition, history, waveform description, and applications. Sensors.

[120] Brüser C., Winter S., Leonhardt S. (2013). Robust inter-beat interval estimation in cardiac vibration signals. Physiol. Meas..

[121] Alivar A., Carlson C., Suliman A., Warren S., Prakash P., Thompson D.E., Natarajan B. (2019). Motion artifact detection and reduction in bed-based ballistocardiogram. IEEE Access.

[122] Shin J., Choi B., Lim Y., Jeong D., Park K. Automatic ballistocardiogram (BCG) beat detection using a template matching approach. Proceedings of the 2008 30th Annual International Conference of the IEEE Engineering in Medicine and Biology Society.

[123] Lydon K., Su B.Y., Rosales L., Enayati M., Ho K., Rantz M., Skubic M. Robust heartbeat detection from in-home ballistocardiogram signals of older adults using a bed sensor. Proceedings of the 2015 37th Annual International Conference of the IEEE Engineering in Medicine and Biology Society (EMBC).

[124] Inan O.T., Etemadi M., Wiard R.M., Giovangrandi L., Kovacs G. (2009). Robust ballistocardiogram acquisition for home monitoring. Physiol. Meas..

[125] Niizeki K., Nishidate I., Uchida K., Kuwahara M. (2005). Unconstrained cardiorespiratory and body movement monitoring system for home care. Med. Biol. Eng. Comput..

[126] Katz Y., Karasik R., Shinar Z. Contact-free piezo electric sensor used for real-time analysis of inter beat interval series. Proceedings of the 2016 Computing in Cardiology Conference (CinC). IEEE.

[127] Hossein A., Rabineau J., Gorlier D., Del Rio J.I.J., Van De Borne P., Migeotte P.F., Nonclercq A. (2021). Kinocardiography derived from ballistocardiography and seismocardiography shows high repeatability in healthy subjects. Sensors.

[128] Taebi A., Solar B.E., Bomar A.J., Sandler R.H., Mansy H.A. (2019). Recent advances in seismocardiography. Vibration.

[129] Tadi M.J., Lehtonen E., Lahdenoja O., Pankaala M., Koivisto T. An adaptive approach for heartbeat detection based on S-transform in seismocardiograms. Proceedings of the 2016 38th Annual International Conference of the IEEE Engineering in Medicine and Biology Society (EMBC).

[130] Tadi M.J., Lehtonen E., Pankäälä M., Saraste A., Vasankari T., Terás M., Koivisto T. Gyrocardiography: A new non-invasive approach in the study of mechanical motions of the heart. Concept, method and initial observations. Proceedings of the 2016 38th Annual International Conference of the IEEE Engineering in Medicine and Biology Society (EMBC).

[131] Wahlström J., Skog I., Händel P., Khosrow-Khavar F., Tavakolian K., Stein P.K., Nehorai A. (2017). A hidden markov model for seismocardiography. IEEE Trans. Biomed. Eng..

[132] Gardner M., Randhawa S., Reynolds K.J., Malouf G. Estimation of heart rate during sleep measured from a gyroscope embedded in a CPAP mask. Proceedings of the 2016 IEEE EMBS Conference on Biomedical Engineering and Sciences (IECBES).

[133] Yu S., Liu S. (2020). A novel adaptive recursive least squares filter to remove the motion artifact in seismocardiography. Sensors.

[134] Yang C., Tavassolian N. Motion noise cancellation in seismocardiogram of ambulant subjects with dual sensors. Proceedings of the 2016 38th Annual International Conference of the IEEE Engineering in Medicine and Biology Society (EMBC).

[135] Hsu P.Y., Hsu P.H., Lee T.H., Liu H.L. Heart Rate and Respiratory Rate Monitoring Using Seismocardiography. Proceedings of the 2021 43rd Annual International Conference of the IEEE Engineering in Medicine & Biology Society (EMBC).

[136] García-González M.A., Argelagós-Palau A., Fernández-Chimeno M., Ramos-Castro J. A comparison of heartbeat detectors for the seismocardiogram. Proceedings of the Computing in Cardiology 2013.

[137] Brüser C., Stadlthanner K., Brauers A., Leonhardt S. Applying machine learning to detect individual heart beats in ballistocardiograms. Proceedings of the 2010 Annual International Conference of the IEEE Engineering in Medicine and Biology.

[138] Mora N., Cocconcelli F., Matrella G., Ciampolini P. (2020). Detection and analysis of heartbeats in seismocardiogram signals. Sensors.

[139] Yang C., Tavassolian N. (2018). An independent component analysis approach to motion noise cancelation of cardio-mechanical signals. IEEE Trans. Biomed. Eng..

[140] Inan O.T., Migeotte P.F., Park K.S., Etemadi M., Tavakolian K., Casanella R., Zanetti J., Tank J., Funtova I., Prisk G.K. (2014). Ballistocardiography and seismocardiography: A review of recent advances. IEEE J. Biomed. Health Informatics.

[141] Lee H., Lee H., Whang M. (2018). An enhanced method to estimate heart rate from seismocardiography via ensemble averaging of body movements at six degrees of freedom. Sensors.

[142] Javaid A.Q., Ashouri H., Dorier A., Etemadi M., Heller J.A., Roy S., Inan O.T. (2016). Quantifying and reducing motion artifacts in wearable seismocardiogram measurements during walking to assess left ventricular health. IEEE Trans. Biomed. Eng..

[143] Wiard R.M., Inan O.T., Argyres B., Etemadi M., Kovacs G.T., Giovangrandi L. (2011). Automatic detection of motion artifacts in the ballistocardiogram measured on a modified bathroom scale. Med. Biol. Eng. Comput..

[144] Cao X., Wang J., Zhang X., Tang J. (2014). A ballistocardiogram measurement system for home monitoring: Design, performance, and evaluation. Chin. Sci. Bull..

[145] Ashouri H., Inan O.T. (2017). Automatic detection of seismocardiogram sensor misplacement for robust pre-ejection period estimation in unsupervised settings. IEEE Sensors J..

[146] Di Rienzo M., Vaini E., Castiglioni P., Merati G., Meriggi P., Parati G., Faini A., Rizzo F. (2013). Wearable seismocardiography: Towards a beat-by-beat assessment of cardiac mechanics in ambulant subjects. Auton. Neurosci..

[147] Javaid A.Q., Ashouri H., Inan O.T. Estimating systolic time intervals during walking using wearable ballistocardiography. Proceedings of the 2016 IEEE-EMBS International Conference on Biomedical and Health Informatics (BHI).

[148] Malešević N., Petrović V., Belić M., Antfolk C., Mihajlović V., Janković M. (2020). Contactless Real-Time Heartbeat Detection via 24 GHz Continuous-Wave Doppler Radar Using Artificial Neural Networks. Sensors.

[149] Kuo H.C., Chou C.C., Lin C.C., Yu C.H., Huang T.H., Chuang H.R. A 60-GHz CMOS direct-conversion Doppler radar RF sensor with clutter canceller for single-antenna noncontact human vital-signs detection. Proceedings of the 2015 IEEE Radio Frequency Integrated Circuits Symposium (RFIC).

[150] Wang Y., Wang W., Zhou M., Ren A., Tian Z. (2020). Remote Monitoring of Human Vital Signs Based on 77-GHz mm-Wave FMCW Radar. Sensors.

[151] Marchionni P., Scalise L., Ercoli I., Tomasini E. (2013). An optical measurement method for the simultaneous assessment of respiration and heart rates in preterm infants. Rev. Sci. Instruments.

[152] Sirevaag E.J., Casaccia S., Richter E.A., O’Sullivan J.A., Scalise L., Rohrbaugh J.W. (2016). Cardiorespiratory interactions: Noncontact assessment using laser Doppler vibrometry. Psychophysiology.

[153] Jeger-Madiot N., Gateau J., Fink M., Ing R.K. (2017). Non-contact and through-clothing measurement of the heart rate using ultrasound vibrocardiography. Med. Eng. Phys..

[154] Kon S., Oldham K., Horowitz R. (2007). Piezoresistive and piezoelectric MEMS strain sensors for vibration detection. Proc. SPIE.

[155] Dainty J.C. (2013). Laser Speckle and Related Phenomena.

[156] Taebi A. (2018). Characterization, Classification, and Genesis of Seismocardiographic Signals. Ph.D. Thesis.

[157] Campo A., Segers P., Heuten H., Goovaerts I., Ennekens G., Vrints C., Baets R., Dirckx J. (2014). Non-invasive technique for assessment of vascular wall stiffness using laser Doppler vibrometry. Meas. Sci. Technol..

[158] Li Y., Segers P., Dirckx J., Baets R. (2013). On-chip laser Doppler vibrometer for arterial pulse wave velocity measurement. Biomed. Opt. Express.

[159] Hayashi T., Hirata S., Hachiya H. (2019). A method for the non-contact measurement of two-dimensional displacement of chest surface by breathing and heartbeat using an airborne ultrasound. Jpn. J. Appl. Phys..

[160] Zalevsky Z., Beiderman Y., Margalit I., Gingold S., Teicher M., Mico V., Garcia J. (2009). Simultaneous remote extraction of multiple speech sources and heart beats from secondary speckles pattern. Opt. Express.

[161] Ozana N., Margalith I., Beiderman Y., Kunin M., Campino G.A., Gerasi R., Garcia J., Mico V., Zalevsky Z. (2015). Demonstration of a remote optical measurement configuration that correlates with breathing, heart rate, pulse pressure, blood coagulation, and blood oxygenation. Proc. IEEE.

[162] Havakuk O., Sadeh B., Merdler I., Zalevsky Z., Garcia-Monreal J., Polani S., Arbel Y. (2021). Validation of a novel contact-free heart and respiratory rate monitor. J. Med. Eng. Technol..

[163] Reyes B.A., Posada-Quintero H.F., Bales J.R., Clement A.L., Pins G.D., Swiston A., Riistama J., Florian J.P., Shykoff B., Qin M. (2014). Novel electrodes for underwater ECG monitoring. IEEE Trans. Biomed. Eng..

[164] Sokas D., Petrėnas A., Daukantas S., Rapalis A., Paliakaitė B., Marozas V. (2019). Estimation of heart rate recovery after stair climbing using a wrist-worn device. Sensors.

[165] Sandau K.E., Funk M., Auerbach A., Barsness G.W., Blum K., Cvach M., Lampert R., May J.L., McDaniel G.M., Perez M.V. (2017). Update to practice standards for electrocardiographic monitoring in hospital settings: A scientific statement from the American Heart Association. Circulation.

[166] McDuff D., Gontarek S., Picard R.W. (2014). Improvements in remote cardiopulmonary measurement using a five band digital camera. IEEE Trans. Biomed. Eng..

[167] Raghuram M., Sivani K., Reddy K.A. Use of complex EMD generated noise reference for adaptive reduction of motion artifacts from PPG signals. Proceedings of the 2016 International Conference on Electrical, Electronics, and Optimization Techniques (ICEEOT).

[168] Lee J., Shin S.S. (2014). Reducing motion artifacts from PPG signals using adaptive threshold algorithm. Int. J. Appl. Eng. Res..

[169] Sun X., Su F., Chen X., Peng Q., Luo X., Hao X. (2020). Doppler ultrasound and photoplethysmographic assessment for identifying pregnancy-induced hypertension. Exp. Ther. Med..

[170] Zhang Z., Pi Z., Liu B. (2014). TROIKA: A general framework for heart rate monitoring using wrist-type photoplethysmographic signals during intensive physical exercise. IEEE Trans. Biomed. Eng..

[171] Temko A. (2017). Accurate heart rate monitoring during physical exercises using PPG. IEEE Trans. Biomed. Eng..

[172] Biswas D., Everson L., Liu M., Panwar M., Verhoef B.E., Patki S., Kim C.H., Acharyya A., Van Hoof C., Konijnenburg M. (2019). CorNET: Deep learning framework for PPG-based heart rate estimation and biometric identification in ambulant environment. IEEE Trans. Biomed. Circuits Syst..

[173] Jarchi D., Casson A.J. (2017). Towards photoplethysmography-based estimation of instantaneous heart rate during physical activity. IEEE Trans. Biomed. Eng..

[174] Marsili I.A., Biasiolli L., Masè M., Adami A., Andrighetti A.O., Ravelli F., Nollo G. (2020). Implementation and validation of real-time algorithms for atrial fibrillation detection on a wearable ECG device. Comput. Biol. Med..

[175] Sološenko A., Petrėnas A., Paliakaitė B., Sörnmo L., Marozas V. (2019). Detection of atrial fibrillation using a wrist-worn device. Physiol. Meas..

[176] Yang C., Veiga C., Rodriguez-Andina J.J., Farina J., Iniguez A., Yin S. (2019). Using PPG signals and wearable devices for atrial fibrillation screening. IEEE Trans. Ind. Electron..

[177] Hurnanen T., Lehtonen E., Tadi M.J., Kuusela T., Kiviniemi T., Saraste A., Vasankari T., Airaksinen J., Koivisto T., Pänkäälä M. (2016). Automated detection of atrial fibrillation based on time–frequency analysis of seismocardiograms. IEEE J. Biomed. Health Informatics.

[178] Eerikäinen L.M., Bonomi A.G., Dekker L.R., Vullings R., Aarts R.M. (2020). Atrial fibrillation monitoring with wrist-worn photoplethysmography-based wearables: State-of-the-art review. Cardiovasc. Digit. Health J..

